# Supplementary figures and images for: Caspase-1-licensed pyroptosis drives dsRNA-mediated necroptosis and dampens host defense against bacterial pneumonia
Source: PLoS Pathog. 2025 May 13;21(5):e1013167. doi: 10.1371/journal.ppat.1013167 (PMC12121916; doi:10.1371/journal.ppat.1013167)

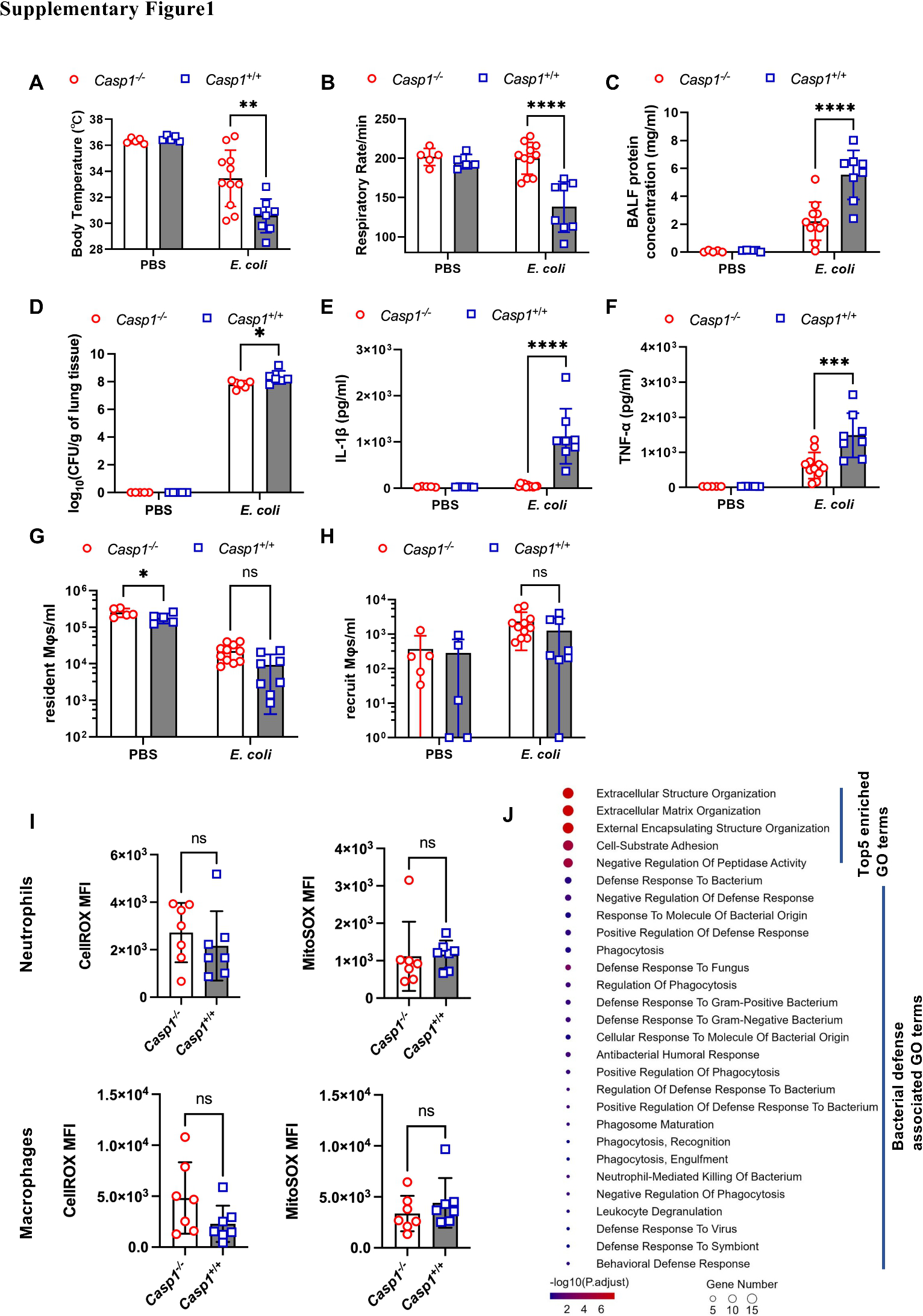

Supplement: S1 Fig — (A and B) Respiratory rates and body temperatures of Casp1-/- mice (n = 11) and Casp1+/+ mice (n = 8) at 12 hours after E. coli infection or intratracheal PBS instillation (n = 5 each). (C) BALF protein concentrations in Casp1-/- mice (n = 11) and Casp1+/+ mice (n = 8) at 12 hours after E. coli infection or intratracheal PBS instillation (n = 5 each). (D) BALF bacterial burden in Casp1-/- mice (n = 11) and Casp1+/+ mice (n = 8) at 12 hours after E. coli infection or intratracheal PBS instillation (n = 5 each) was determined and is expressed as CFU/ml. (E and F) IL-1β and TNF-α levels in the BALF of Casp1-/- mice (n = 11) and Casp1+/+ mice (n = 8) at 12 hours after E. coli infection or intratracheal PBS instillation (n = 5 each) were measured by ELISAs. (G and H) Resident macrophages (SiglecF+Ly6C-F4/80+) and recruited monocyte (SiglecF-Ly6C+F4/80+) in the BALF of Casp1-/- mice (n = 11) and Casp1+/+ mice (n = 8) at 12 hours after E. coli infection or intratracheal PBS instillation (n = 5 each) were counted by flow cytometry. (I) Total cellular ROS (determined by CellROX) and mitochondrial ROS (determined by MitoSOX staining) in neutrophils and macrophages from the BALF of Casp1-/- mice and Casp1+/+ mice at 12 hours after pulmonary E. coli infection were analyzed by flow cytometry (n = 7). (J) Pathway enrichment of expressed genes in neutrophils from the BALF of Casp1-/- mice and Casp1+/+ neutrophils at 12 hours after pulmonary E. coli infection was analyzed using RNA-seq transcriptomes (n = 2). The data are shown as the means ± SDs (A-I). Statistical differences were determined by two-way ANOVA (A‒H), Student’s t test (I) and the parametric F test comparing nested linear models (J). *P < 0.05; **P < 0.01; ***P < 0.001; ****P < 0.0001. ns, not significant. CFU, colony-forming unit. (TIF) [file ppat.1013167.s001.tif]

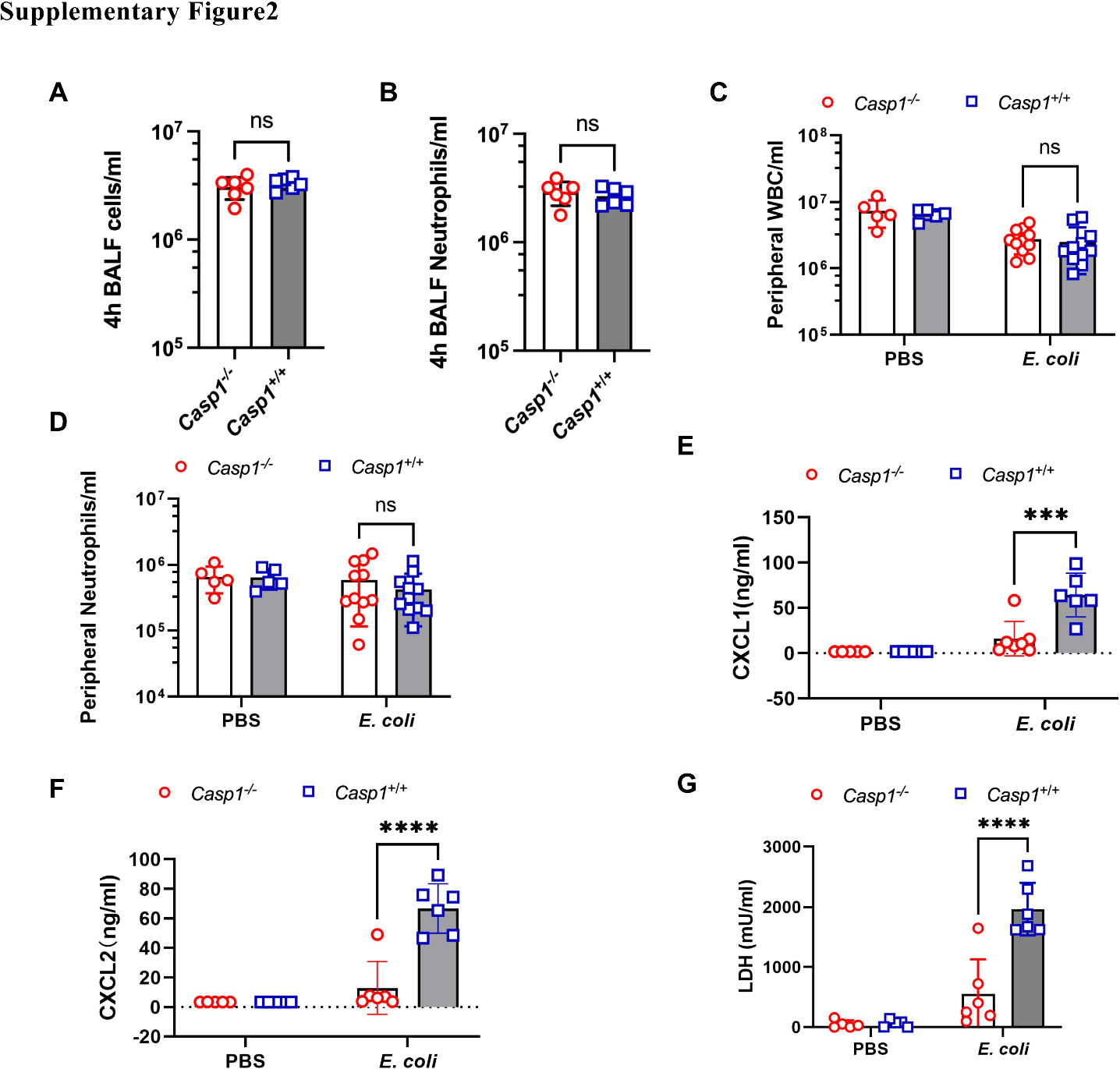

Supplement: S2 Fig — BALF cells (A) and BALF neutrophils (B) from Casp1-/- mice (n = 6) and Casp1+/+ mice (n = 6) were counted at 4 hours after bacterial instillation. Peripheral white blood cells (C) and neutrophils (D) from Casp1-/- mice and Casp1+/+ mice were counted at 12 hours after E. coli challenge (n = 11 each) or PBS instillation (n = 5 each). BALF levels of CXCL1 (E) and CXCL2 (F) in Casp1-/- mice and Casp1+/+ mice were measured at 12 hours after E. coli challenge (n = 11 each) or PBS instillation (n = 5 each). (G) LDH levels in the BALF of Casp1-/- mice and Casp1+/+ mice at 12 hours after E. coli infection (n = 6) or intratracheal PBS instillation (n = 5). The data are shown as the means ± SDs in (A-G). Statistical differences were determined by Student’s t test (A and B) and one-way ANOVA (C, D, E, F and G). ***P < 0.001; ****P < 0.0001. ns, not significant. (TIF) [file ppat.1013167.s002.tif]

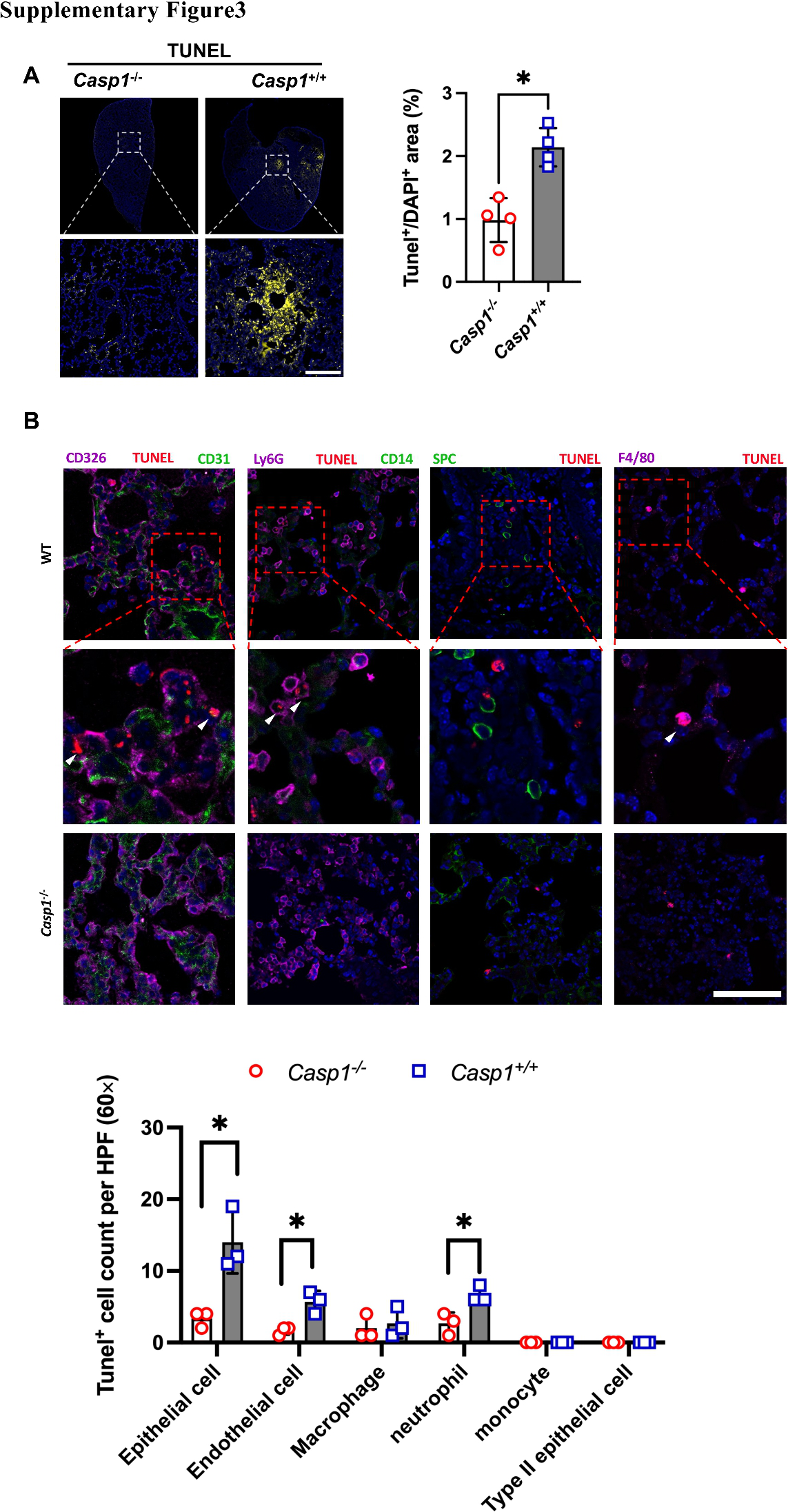

Supplement: S3 Fig — (A) TUNEL staining of lung sections from Casp1-/- mice and Casp1+/+ mice at 12 hours after E. coli infection and quantification of the TUNEL-positive area relative to the DAPI-stained area (n = 4). Scale bars, 100 μm. (B) Casp1+/+ and Casp1-/- mouse lungs were harvested at 12 hours after E. coli infection. TUNEL staining of lung sections was performed with different markers including the epithelial marker CD326, the endothelial marker CD31, the type II epithelial marker SPC, the macrophage marker F4/80, the neutrophil marker Ly-6G, and the monocyte marker CD14. Quantification of the percentage of TUNEL+ cells of different cell type (n = 3 biologically independent samples). Scale bars, 50 μm. The data are shown as the means ± SDs and are representative of 3 independent experiments. Statistical differences were determined by Student’s t test. *P < 0.05. HPF, high-power field. (TIF) [file ppat.1013167.s003.tif]

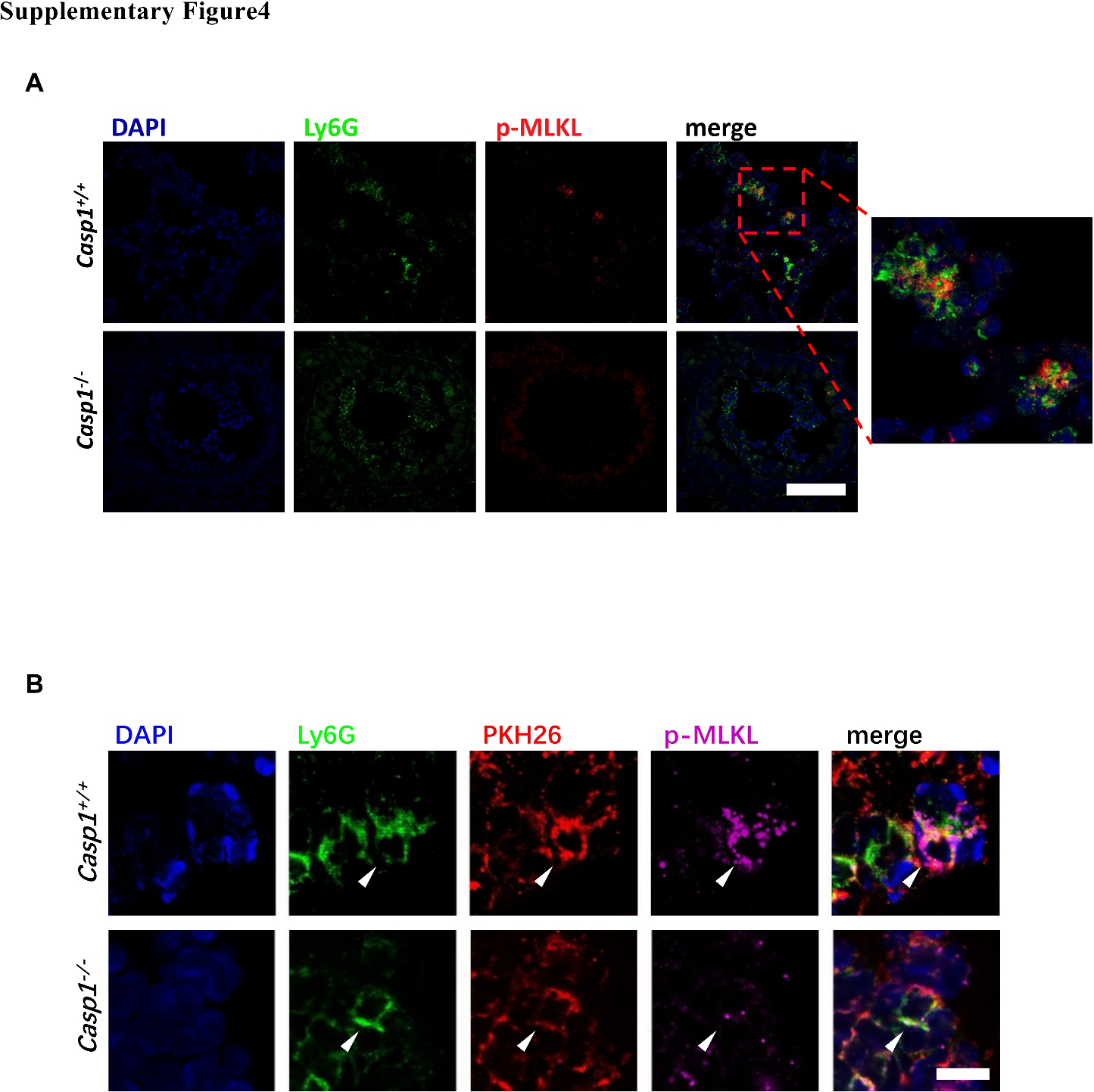

Supplement: S4 Fig — (A) Casp1+/+ and Casp1-/- mouse lungs were harvested at 12 hours after E. coli infection. p-MLKL staining of lung sections with the neutrophil marker Ly6G was performed by immunofluorescence. Scale bars, 50 μm. (B) BALF cells from Casp1-/- mice and Casp1+/+ mice were fixed on slides, stained with PKH26 and p-MLKL antibodies and visualized by using confocal microscopy. Scale bars, 10 μm. The data are representative of 3 independent experiments. (TIF) [file ppat.1013167.s004.tif]

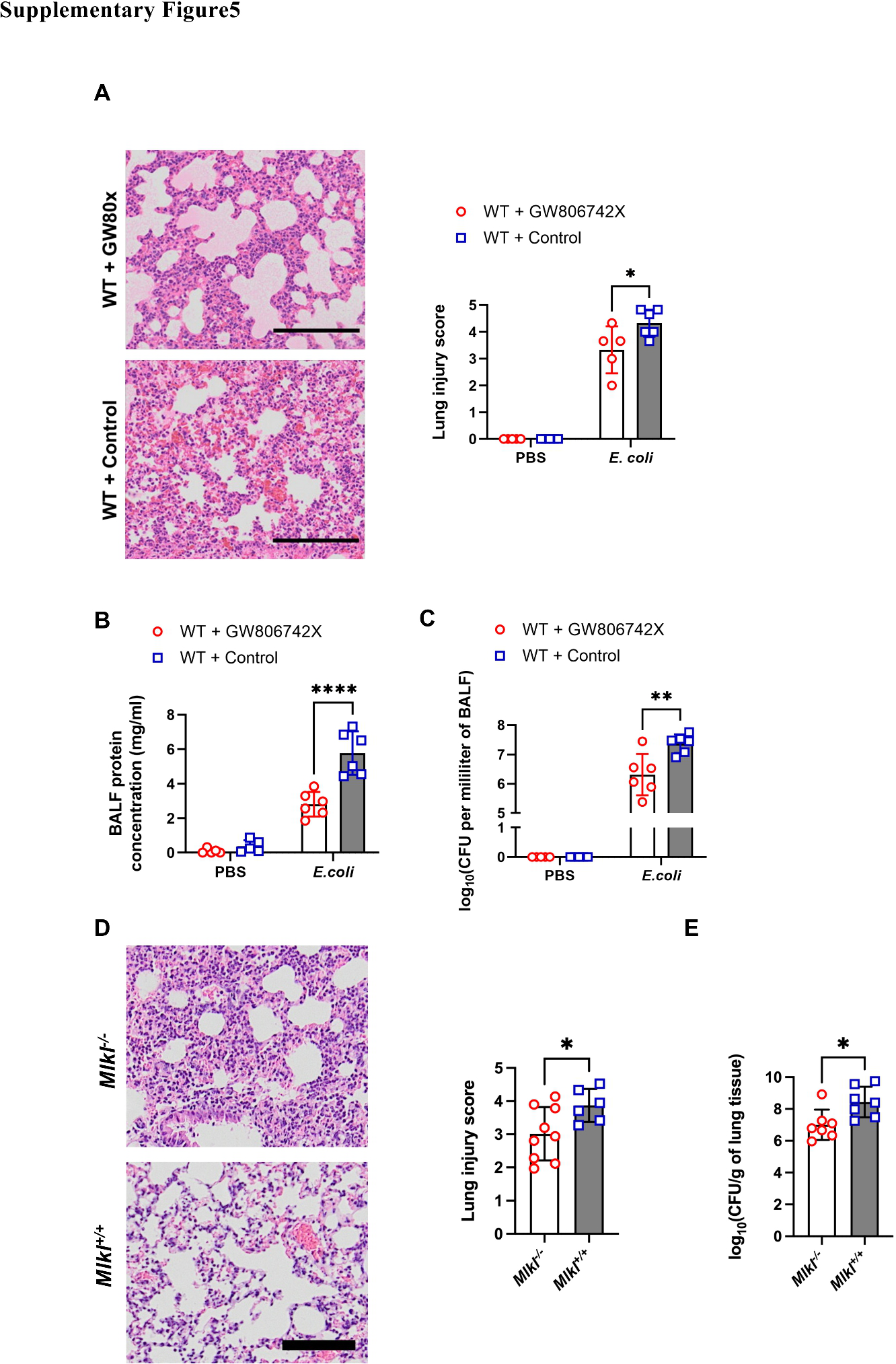

Supplement: S5 Fig — (A) Representative images of lungs with H&E staining and quantification of lung injury scores in GW806742X (n = 5)- or control solvent (n = 6)-treated WT mice at 12 hours after pulmonary E. coli infection or intratracheal PBS instillation (n = 5 each). Scale bars, 100 µm. BALF protein concentration (B) and bacterial burden (C) in WT mice treated with GW806742X (n = 6) or control solvent (n = 6) at 12 hours after pulmonary E. coli infection or intratracheal PBS instillation (n = 5 each). (D) Representative images of lungs with HE staining and quantification of lung injury scores in Mlkl-/- (n = 9) or Mlkl+/+ (n = 6) mice at 12 hours after pulmonary E. coli infection. Scale bars, 100 µm. (E) Twelve hours after E. coli infection, mouse lungs were harvested from Mlkl-/- or Mlkl+/+ mice, and the bacterial burdens in whole-lung tissue were determined. The data are shown as the means ± SDs (A, B, C, D and E). Statistical differences were determined by Student’s t test (D and E) and two-way ANOVA (A, B and C). *P < 0.05; **P < 0.01; ****P < 0.0001. CFU, colony-forming unit. (TIF) [file ppat.1013167.s005.tif]

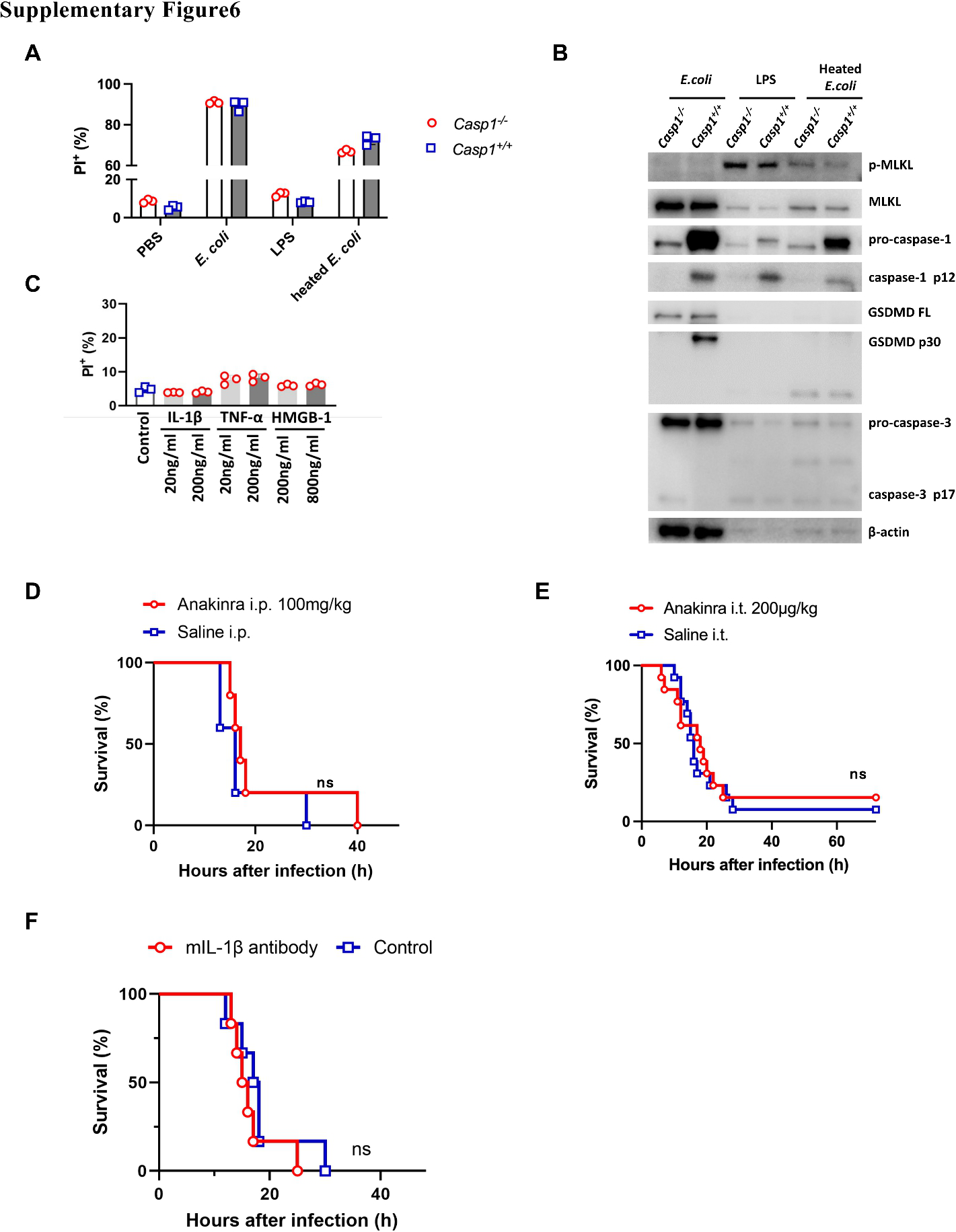

Supplement: S6 Fig — (A) Proportion of PI+ neutrophils from Casp1-/- and Casp1+/+ mice after stimulation with LPS, live E. coli, heat-inactivated E. coli or PBS (n = 3). (B) Immunoblot analysis of cell death-related proteins in neutrophils stimulated with LPS, live E. coli or heat-inactivated E. coli. (C) Proportion of PI+ neutrophils from WT mice treated with IL-1β, TNF-α, HMGB-1 or control solvent at the indicated concentrations (n = 3). (D and E) Survival of WT mice intraperitoneally (n = 5 each) or intratracheally (n = 6 each) pretreated with the IL-1β antagonist anakinra or saline after pulmonary E. coli infection. (F) Survival of WT mice intraperitoneally (n = 6 each) pretreated with the anti-IL-1β antibody or control IgG antibody after pulmonary E. coli infection. The data are shown as the means ± SDs in (A and C) and are representative of 3 independent experiments in (B). Statistical differences were determined by the Mantel‒Cox test (D, E and F). ns, not significant. (TIF) [file ppat.1013167.s006.tif]

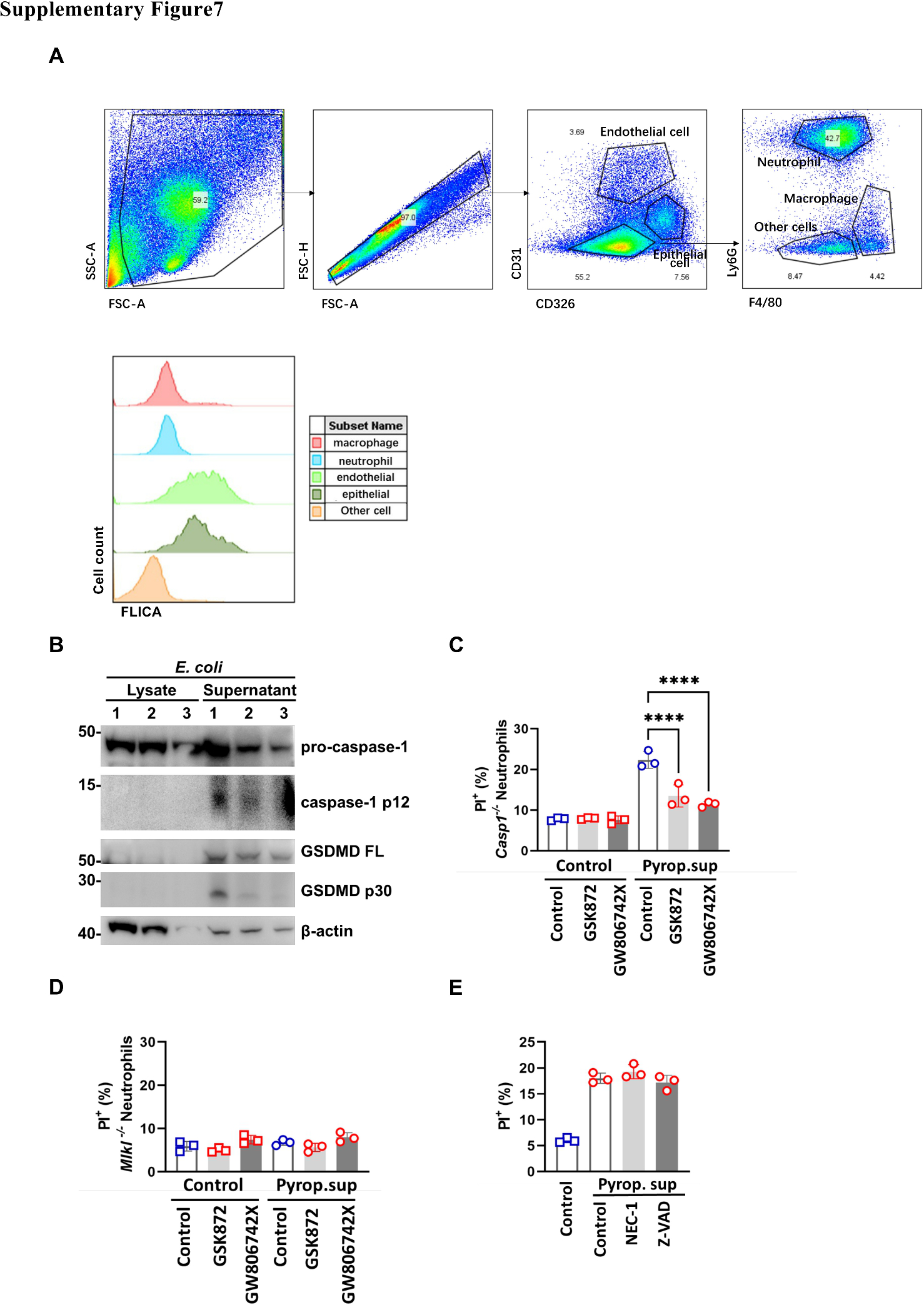

Supplement: S7 Fig — (A) Flow cytometry gating strategy for identifying caspase-1-activated cells in mouse lungs via FLICA staining. (B) MLE-12 epithelial cells were stimulated with E. coli, and then, the cell lysate and culture supernatant were collected and immunoblotted for pyroptosis-related proteins. (C and D) Casp1-/- neutrophils and Mlkl-/- neutrophils were pretreated with GSK872, GW806742X or control solvent and then stimulated with pyroptotic supernatant (pyrop. sup) or control medium, and cell death was assayed by flow cytometry (n = 3). (E) WT neutrophils were pretreated with NEC-1, Z-VAD or control solvent and then stimulated with pyrop. sup or control medium, and cell death was assayed by flow cytometry (n = 3). The data are shown as the means ± SDs in (C, D and E) and are representative of 3 independent experiments in (B). Statistical differences were determined by two-way ANOVA (C and D) or one-way ANOVA (E). ****P < 0.0001. (TIF) [file ppat.1013167.s007.tif]

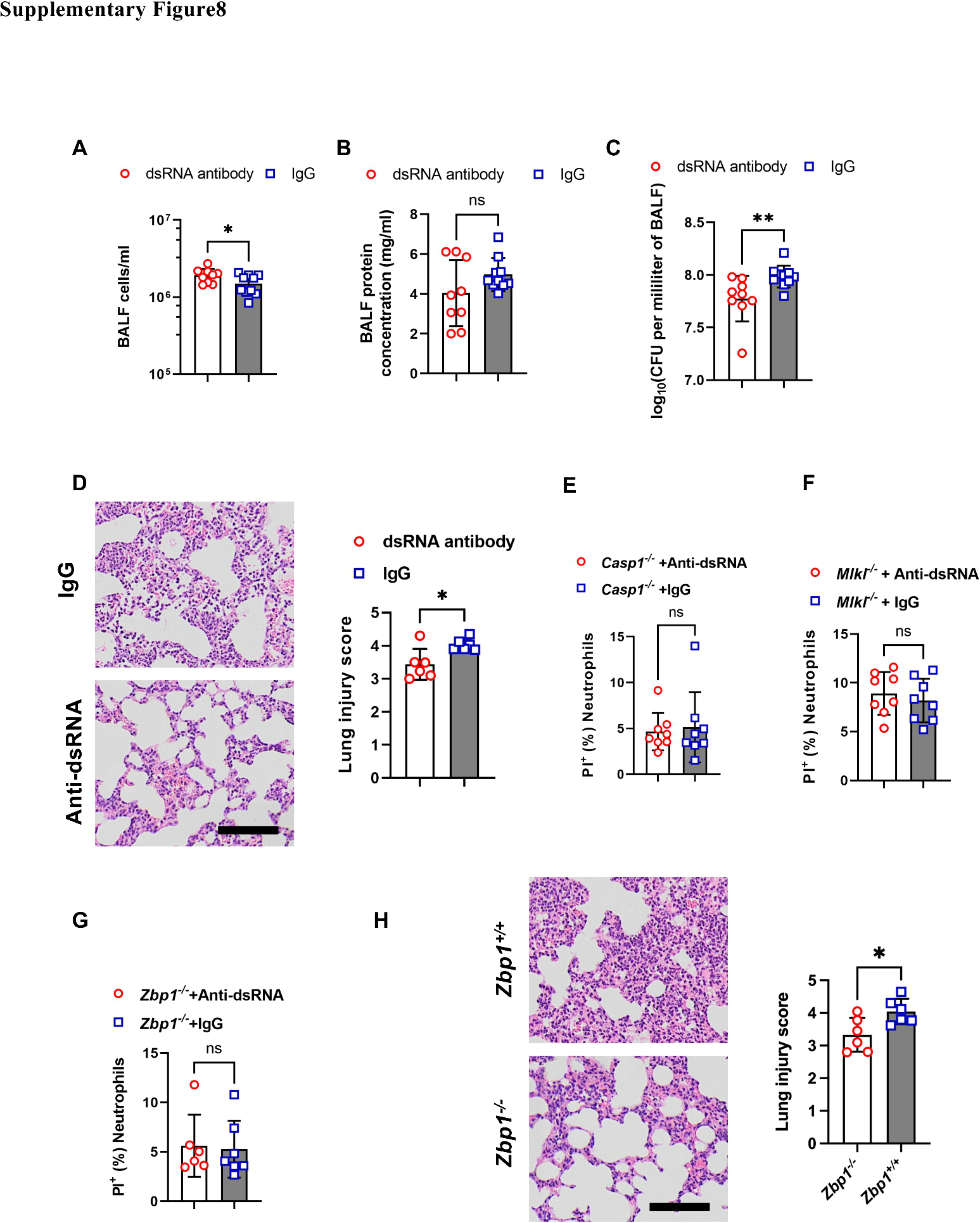

Supplement: S8 Fig — The total BALF cell number (A), protein concentration (B) and bacterial burden (C) were measured in the mice pretreated with the anti-dsRNA antibody (n = 9) or the IgG control antibody (n = 10) at 12 hours after pulmonary E. coli infection. (D) Representative images of lungs with HE staining and quantification of lung injury scores in anti-dsRNA antibody (n = 6)- or control IgG (n = 6)-treated WT mice at 12 hours after pulmonary E. coli infection. Scale bars, 100 µm. (E‒G) Proportion of PI+ neutrophils in the BALF of Casp1-/- (L), Mlkl-/- (M) and Zbp1-/- (N) mice treated with an anti-dsRNA antibody (n = 6‒8) or an IgG control antibody (n = 7‒8) at 12 h after pulmonary E. coli infection. (H) Representative images of lungs with HE staining and quantification of lung injury scores in Zbp1-/- (n = 6) or Zbp1+/+ littermate (n = 6) mice at 12 hours after pulmonary E. coli infection. Scale bars, 100 µm. The data are shown as the means ± SDs. Statistical differences were determined by Student’s t test. *P < 0.05; **P < 0.01. ns, not significant. CFU, colony-forming unit. (TIF) [file ppat.1013167.s008.tif]

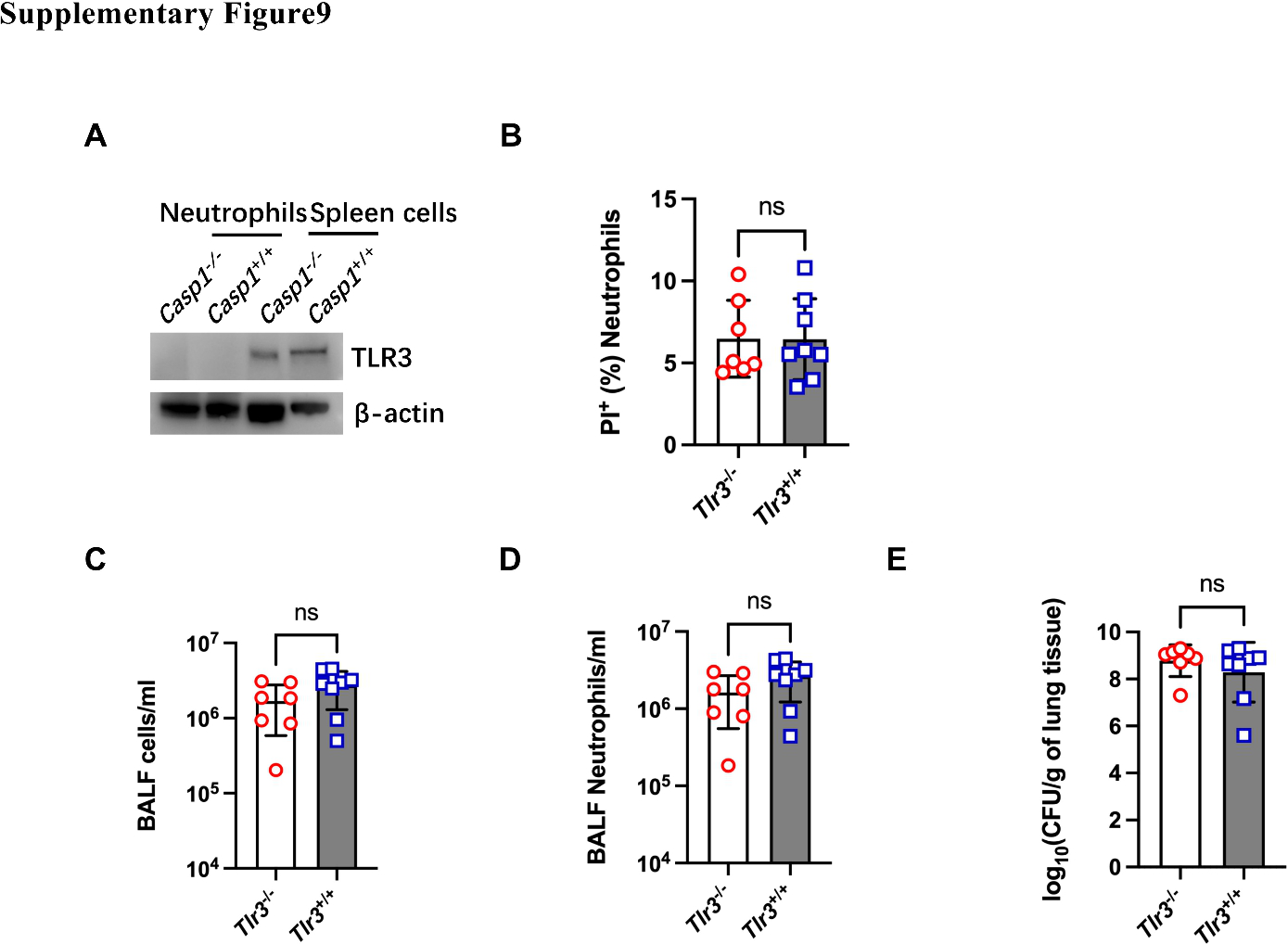

Supplement: S9 Fig — (A) Western blot analysis of BALF neutrophils and spleen cells derived from E. coli-infected Casp1-/- and Casp1+/+ mice at 12 hours. (B-E) Twelve hours after E. coli infection, the proportions of PI+ neutrophils (B), neutrophil numbers (C), total leukocytes (D) and bacterial burdens (E) in the BALF of Tlr3+/+ mice (n = 8) and Tlr3-/- mice (n = 7) were determined. The data are shown as the means ± SDs (B-E) and are representative of 2 independent experiments in (A). Statistical differences were determined by Student’s t test (B-D). ns, not significant. CFU, colony-forming unit. (TIF) [file ppat.1013167.s009.tif]
