# Supplementary material for: Caspase-1-licensed pyroptosis drives dsRNA-mediated necroptosis and dampens host defense against bacterial pneumonia
Source: PLoS Pathog. 2025 May 13;21(5):e1013167. doi: 10.1371/journal.ppat.1013167 (PMC12121916; doi:10.1371/journal.ppat.1013167)
Supplement: Attachment — Submitted filename: point to point response letter1121.pdf [file ppat.1013167.s015.pdf]

Point-by-point responses to reviewers' comments

Reviewer #1:

1. One of the major claims is that Casp1-dependent pyroptosis is primarily occurring in epithelial cells, whereas Zbp1/Ripk3/Mlkl-dependent pyroptosis is primarily occurring in neutrophils downstream of epithelial pyroptosis. As the authors correctly point out, the in vivo experiments do not allow for this strong conclusion.

o Cell-type specific KO or KI mice (e.g., deletion of *Casp1* and Mlkl in epithelial vs neutrophils) to assess these claims would be welcome and definitive, but I recognize the considerable time and cost associated with such experiments. Nevertheless, the authors focus primarily on epithelia, which ignores the result in Fig. 4A and 4B that Casp1 is active in endothelia. Thus, it is unclear whether the in vivo source of pyroptosis (and the presumed release of dsRNA) is epithelial, endothelia, or both.

o Reciprocal bone marrow chimera experiments would be an appropriate alternative. If the authors choose to do so, they might consider also including Gsdmd KO mice since they formally did not genetically demonstrate the role of pyroptosis in vivo or in vitro.

Response: Yes, thank you for the comments and suggestions. According to your suggestion, we conducted the reciprocal bone marrow chimera experiments and monitored survival, neutrophil death and dsRNA release as shown in the following figures. The results indicated that mice with $Casp1^{-/-}$ or $Gsdmd^{-/-}$ non-hematopoietic cells had an improved survival, reduced neutrophil death and dsRNA release (Fig. 4, H and I and Fig. 5E).

Fig. 4H

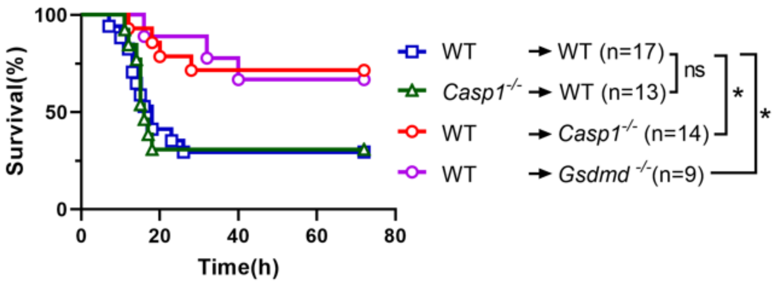

Fig. 4I

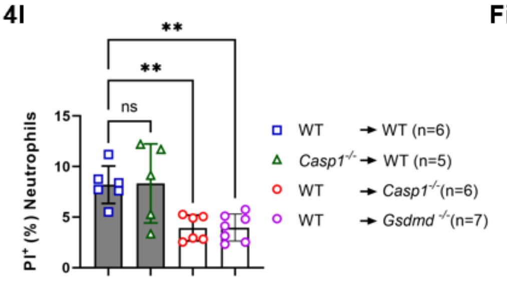

Fig. 5E

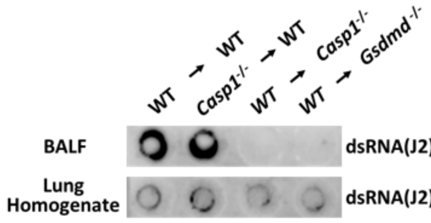

Reciprocal bone marrow chimera experiments.

(Fig. 4, H and I) Chimeric mice were created by reciprocal bone marrow transplantation between WT, *Casp1*$^{-/-}$ and *Gsdmd*$^{-/-}$ mice as indicated, and then mice were subjected to *E.coli*-induced pneumonia. Mouse survival was monitored (H). BALF cells were collected at 12 hours and neutrophil death was determined by PI staining and flow cytometry (I). The survival experiment was conducted across three independent replicates, with each replicate including 3–6 mice.

(Fig. 5E) Dot blot of BALF from indicated chimeric mice at 12 hours after pulmonary *E.coli* infection using anti-dsRNA antibody.

Data are shown as the mean ± SD. Statistical differences were determined by Mantel–Cox test and (4H) one-way ANOVA (4I). *P < 0.05; **P < 0.01. ns, not significant.

o While I find the in vitro experiments with MLE12 and neutrophils a useful in vitro model, the authors could much more rigorously support their claims by genetically

deleting genes relevant to the inflammasome and necroptotic pathways in both MLE12 and neutrophils. Editing primary neutrophils may not be possible, but presumably neutrophils could be used from KO mice in this study, or alternatively, make use of more genetically tractable cell lines (e.g., Cas9+ER-Hoxb8 neutrophil progenitor). Such a model would also allow for complementation of KO neutrophils with variants of interest (e.g., Zbp1 mutants that lack the capacity to bind dsRNA).

Response: Thank you for the suggestion. As you suggested, we employed siRNA to genetically silence caspase-1 and GSDMD in MLE-12 cells. The supernatant collected from inflammasome-silenced MLE-12 cells demonstrated a diminished capacity to induce neutrophil death (Fig. 4, E and F). Additionally, we also used *Mlkl*$^{-/-}$ or *Zbp1*$^{-/-}$ neutrophils in this model. The supernatant obtained from *E.coli*-stimulated pyroptotic epithelial cells resulted in reduced death of *Mlkl*$^{-/-}$ or *Zbp1*$^{-/-}$ neutrophils compared with WT neutrophils (Fig. 6E).

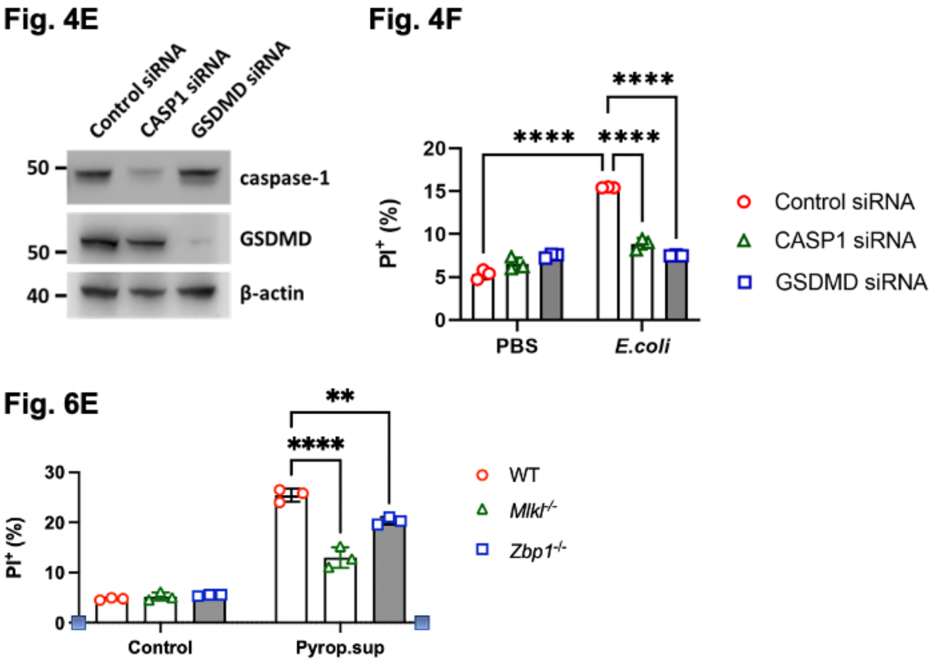

Supernatant collected from inflammasome-silenced MLE-12 cells showed diminished

capacity to induce neutrophil death.

(Fig. 4E) Immunoblot for caspase-1 and GSDMD of MLE-12 cells treated with control siRNA, caspase-1 siRNA or GSDMD siRNA.

(Fig. 4F) Supernatants from PBS or *E.coli* stimulated MLE-12 cells which was pretreated with caspase-1 or GSDMD siRNA were collected and used to stimulate neutrophils, and cell death was evaluated by PI staining and flow cytometry (n = 3).

(Fig. 6E) WT, *Mlkl*$^{-/-}$, and *Zbp1*$^{-/-}$ neutrophils (n=3) were treated with *E.coli* stimulated epithelial cell supernatant (Pyrop.sup) or control medium, cell death was managed by flow cytometry.

Data are shown as the mean ± SD in (4F and 6E) and are representative of 3 independent experiments in (4E). Statistical differences were determined by one-way ANOVA (4F and 6E). **P < 0.01; ****P < 0.0001.

2. The role for dsRNA in neutrophil necroptosis relies primarily on two observations: 1) that Zbp1 KO mice are protected from disease, and 2) injection of a dsRNA binding antibody attenuates disease. This is a remarkable claim, and is the key mechanistic detail linking the two cell death modalities in a non-cell intrinsic manner. Thus, further validation either in vivo or in vitro is important to substantiate the author's conclusions:

o A prediction of the model is that the injection of dsRNA binding Ab would have no effect on a Zbp1, Ripk3, or Mlkl KO mouse, or similarly, in the response of neutrophils from those mice ex vivo.

Response: This is an insightful suggestion and an important validation for dsRNA

related pathway. As we currently do not have access to RIPK3 knockout mice, we chose to intratracheally inject dsRNA antibody into Zbp1 and Mlkl knockout mice before the induction of *E.coli* pneumonia, in accordance with your suggestion. The flow cytometry results indicated that treatment with anti-dsRNA antibody did not reduce neutrophil death in Zbp1 or Mlkl knockout mice (Fig. 5, M and N).

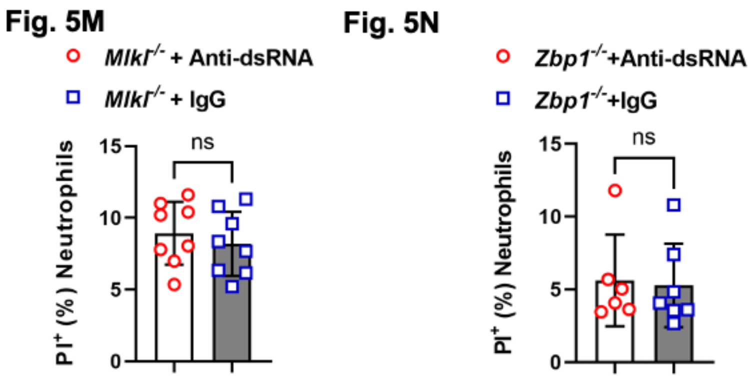

Treatment with anti-dsRNA antibody did not reduce neutrophil death in *Mlkl*$^{-/-}$ and *Zbp1*$^{-/-}$ mice after pulmonary *E.coli* infection.

(Fig. 5, M-N) Proportion of PI$^+$ neutrophils in the BALF of *Mlkl*$^{-/-}$ (L) and *Zbp1*$^{-/-}$ (M) mice treated with anti-dsRNA antibody (n = 6-8) or IgG control antibody (n = 7-8) at 12 h after pulmonary *E.coli* infection.

Data are shown as the means ± SD. Statistical differences were determined by the Student's *t* test. ns, not significant.

o Epithelial cell death is not sufficient to induce neutrophil death, but an explanation is not provided. The authors should present data to determine if dsRNA is released from non-*E.coli* infected epithelia, and if so, if dsRNA is being taken up by neutrophils in the absence of bacteria. Similarly, the experiments in Figure S4A (e.g., WT vs *Casp1*

KO challenged with *E.coli*, heat-killed *E.coli*, etc) should be conducted on MLE12 cells, followed by conditioned supernatants from those studies being used to evaluate necroptosis in neutrophils derived from WT and relevant KO animals. Moreover, the authors rule out a role for IL-1B, but it remains possible that IL-18 or other signals released from pyroptotic epithelia are required to stimulate neutrophils for either the uptake of dsRNA or activation of the necroptotic pathway.

Response: Thank you for your suggestion. We have conducted experiments demonstrating that 1) dsRNA can be released from unstimulated epithelial cells but the quantity is less than *E.coli* stimulated epithelial cells (Fig. 5D); 2) dsRNA interacted neutrophil decreased in the absence of bacteria supernatant (Fig. 5F); 3) In the absence of bacteria supernatant, there is less neutrophil intracellular dsRNA and less ZBP1 colocalized dsRNA (Fig. 5G).

We stimulated the caspase-1 and GSDMD knockdown epithelial cells with live *E.coli* and observed that the conditioned supernatant induced less neutrophil death (Fig. 4F). Similarly, when Mlkl and Zbp1 knockout neutrophils were stimulated with pyroptotic supernatant, they exhibited reduced cell death (Fig. 6E).

Fig. 5D

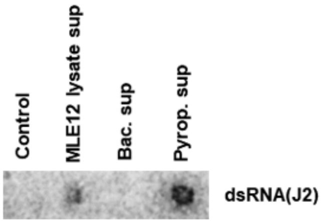

Fig. 5F

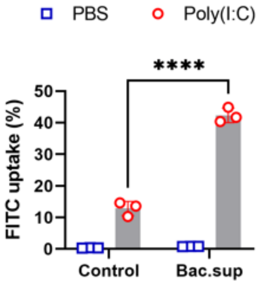

Fig. 5G

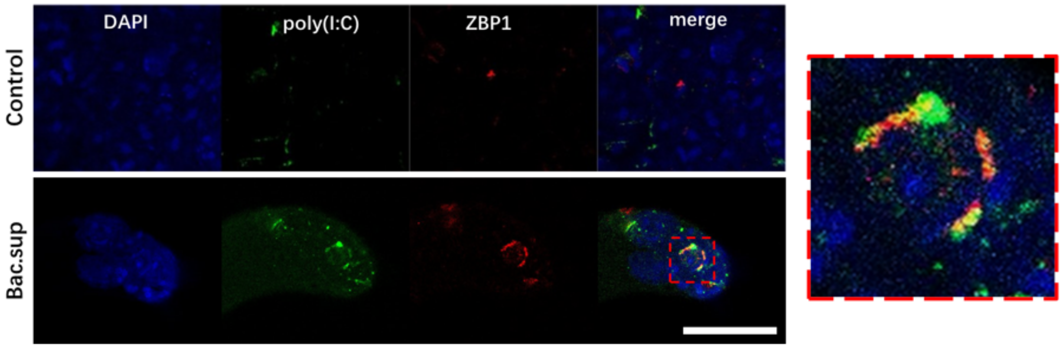

Fig. 4F

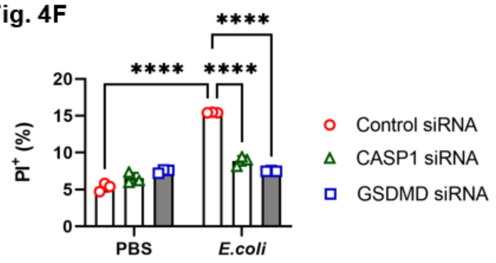

Fig. 6E

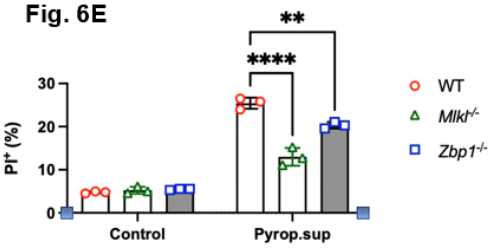

dsRNA is less interacted and uptaken by neutrophils in the absence of bacteria supernatant.

(Fig. 5D) Dot blot of MLE12 lysate supernatant (MLE12 lysate sup), *E.coli* culture supernatant (Bac. sup) and pyroptotic epithelial supernatant (Pyrop. sup) as well as control medium using dsRNA antibody.

(Fig. 5F) Neutrophil interaction of FITC-conjugated poly(I:C) measured by flow cytometry after stimulation with *E.coli* culture supernatant (Bac. Sup) or control medium with/without FITC-conjugated poly(I:C) (n = 3).

(Fig. 5G) Immunofluorescence of neutrophils cocultured with control/Bac. sup and

FITC-conjugated poly(I:C) using anti-ZBP1 antibody. Scale bars, 25 μm.

(Fig. 4F) Supernatants from PBS or *E.coli* stimulated MLE-12 cells pretreated with caspase-1 or GSDMD siRNA were collected and used to stimulate neutrophils, and cell death was evaluated by PI staining and flow cytometry (n = 3).

(Fig. 6E) WT, *Mlkl*$^{-/-}$, and *Zbp1*$^{-/-}$ neutrophils (n=3) were treated with *E.coli* stimulated epithelial cell supernatant (Pyrop.sup) or control medium, cell death was managed by flow cytometry.

Data are shown as the mean ± SD in (5F, 4F and 6E) and are representative of 3 independent experiments in (5D and 5G). Statistical differences were determined by one-way ANOVA (5F, 4F and 6E). **P < 0.01, ****P < 0.0001.

Lastly, we stimulated wild-type neutrophils with various concentrations of IL-18 for 4 hours and cell death is measured by PI staining and flow cytometry. We found that IL-18 alone is insufficient to induce neutrophil death as shown in following figure. Data are shown as the mean ± SD.

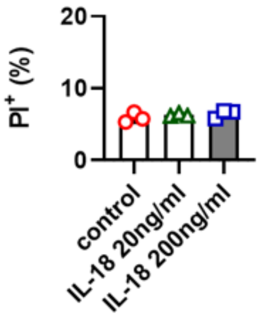

Minor Issues:

Reviewer #1:

1. It is unclear why preventing neutrophil necroptosis limits pathogenesis. Do the authors consider this to be similar to pathology associated with NETosis?

Response: We propose that neutrophil necroptosis may contribute to the overall decrease in neutrophil numbers, which could impair the elimination of pathogens. Necroptosis is validated by our works and reported by others to exacerbate lung bacterial infections (1, 2). NETosis is a regulated form of neutrophil cell death that affected the host defense against pathogens (3). NETosis could exacerbate infective diseases and inhibition of NETosis reduces lung injury in several infective models of lung diseases (4-6). In this aspect, we think neutrophil necroptosis may be a similar pathological process to NETosis.

2. Can the authors provide an explanation based on their model for why *E.coli* CFU is lower in all KOs or interventions that prevent pyroptosis and necroptosis?

Response: Yes. Depletion of neutrophils leads to rapid mortality in infected mice (Fig 1F). When neutrophil deaths are prevented, the number of neutrophils preserved. A sufficient population of neutrophils plays a crucial role in eliminating pathogen invasions.

3. I find it somewhat surprising that Escherichia coli Seattle 1946 strain induces epithelial cell *Casp1*-dependent death. Presumably this strain is attaching/effacing, and not replicating intracellularly? Can the authors comment on this and the nature of the upstream sensor that induces *Casp1* activation in lung epithelia?

Response: Yes, thank you for the comments. *E.coli* ATCC 25922 has been reported to induce caspase-1-dependent pyroptosis in our study and others (7-9). We have also validated that *E.coli* 25922 elicits active caspase-1 and GSDMD both *in vivo* and *in vitro* (Fig. 2D and Fig. S5A). However, the precise mechanism by which this strain induces pyroptosis remains unclear. It is possible that this strain adheres to epithelial cells and other non-hematopoietic cells and disrupts their integrity, thereby activating inflammasomes such as NLRP3 or NLRC4, which sense bacterial LPS, flagellin, or bacterial toxins (10, 11).

4. Similarly, while I do not think it is within the scope of the present work, it would be interesting to know the inflammasome-forming sensor upstream of *Casp1*. This would increase the impact of the manuscript.

Response: Yes, this is a good suggestion. Understanding the upstream inflammasomes that activate caspase-1 is another important aspect of the work. However, our primary focus has been on the downstream effects of caspase-1 activation during *E.coli* pneumonia. We have included this insight into our discussion section in revised manuscript in line 434-437: "Third, the initiating inflammasome upstream of caspase-1 in this model has not been investigated. Inflammasomes are crucial activators of caspase-1 and pyroptosis. Understanding this mechanism could provide valuable insights for manipulating the pathophysiology of bacterial pneumonia." We appreciate your understanding and will consider this suggestion for future research.

5. Tracheal infection of *E.coli* is not very physiologic. Demonstrating this pathway occurs in the context of a respiratory pathogen would increase relevance.

Response: Yes, this is a good suggestion. *E.coli* is a common cause of nosocomial pneumonia, particularly in patients with ventilators, and it frequently affects immunocompromised individuals with underlying health conditions or weakened immune systems (12, 13). We acknowledge that utilizing a respiratory pathogen would enhance the relevance of our findings. We additionally employed a clinical isolated *E.coli* strain in this model, the result showed that caspase-1 knockout also lower the death of neutrophils (Fig. 2B). We appreciate your understanding and will include your suggestions for future research.

Fig. 2B

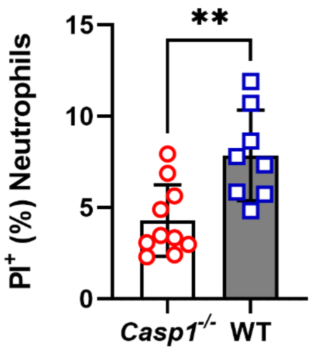

Caspase-1 knockout decreased alveolar neutrophil death in lung infected with a clinical isolated *E.coli* strain.

(Fig. 2B) Neutrophil death in the BALF of *Casp1*$^{-/-}$ and WT mice was measured at 12 hours post-infection with a clinical isolate *E.coli* (n = 8-10). Cell viability was measured by PI staining and analyzed using flow cytometry.

Data are shown as the mean ± SD. Statistical differences were determined by the Mann–Whitney U test. **$P < 0.01$.

6. In Fig. S4B, *Casp1* does seem to be active in neutrophils upon challenge with *E.coli* or LPS. Can the authors speak to this?

Response: Yes, we observed that caspase-1 is activated in neutrophils when directly stimulated with LPS or *E.coli*. However, this phenomenon differs from what we observed *in vivo* (Fig. 2D), where BALF cells exhibited no activated caspase-1. The difference between *in vitro* experiment and *in vivo* experiment may result from the difference in these two models. In mice model there is no neutrophil in the normal lung and after bacteria invading neutrophils were recruited into the lung. So bacteria may first attacked the non-hematopoietic cells and then the non-hematopoietic released component induced neutrophil necroptosis. So we used another model with *E.coli* stimulated epithelial supernatant to induce neutrophil necroptosis *in vitro*.

7. For each mouse experiment, please clarify if mice were littermates, cohoused, or neither.

Response: Thank you for your kind reminder. We have clarified this in our revised manuscript in the first section of Materials/Subjects as line 465-467: "Experiments involving gene-edited mice were performed with littermate controls, while experiments involving antibody or drug treatments were conducted using purchased, co-housed mice controls."

Reviewer #2:

1. While *E.coli* pneumonia can occur, the strain used, ATCC 25922, is a non-pathogen. A relevant virulent clone known to cause pulmonary disease in humans or experimental models should be used.

Response: Yes, thank you for your valuable suggestion. We acknowledge that ATCC 25922 is a non-pathogenic strain and appreciate your suggestion to utilize a more virulent clone. We additionally employed a clinical isolated *E.coli* strain in this model, and the result showed that caspase-1 knockout also lower the death of neutrophils (Fig. 2B). We appreciate your understanding and will consider your suggestions for future study.

Fig. 2B

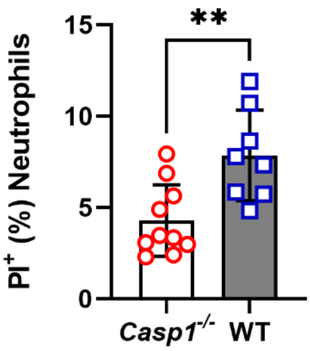

Caspase-1 knockout decreased alveolar neutrophil death in lung infected with a clinical isolated *E.coli* strain.

(Fig. 2B) Neutrophil death in the BALF of *Casp1*$^{-/-}$ and WT mice was measured at 12 hours post-infection with an *E.coli* clinical isolate (n = 8-10). Cell viability was measured by PI staining and analyzed using flow cytometry.

Data are shown as the mean ± SD. Statistical differences were determined by the Student’s *t* test. **P < 0.01.

2. Furthermore, there are no counts of bacteria in the lung for nearly experiment. This is critical to include throughout, and is very unconventional to exclude. A major limitation to the interpretation that the authors should additionally discuss how the greater survival of *Casp1*$^{-/-}$ mice, and their lessor injury, could be due to them just having fewer bacteria (Fig S1). In this sense, inflammatory responses are typically proportional to bacterial load, and the effects reported could be entirely a consequence of that. Effects on IL-1b, respiration, temperature, neutrophil number, and their necroptosis, would all expect to be consequent to that, and not necessarily through the described casp-1-dependent mechanism.

Response: Yes, thank you for the suggestion. We utilized bacterial counts from bronchoalveolar lavage fluid (BALF) instead of whole lung tissue, a method widely employed to demonstrate bacterial clearance in lung infection models (14-16). Based on our methods, the volume of PBS injected into each mouse (0.5 ml × 3, totaling 1.5 ml) and the volume extracted (1.2–1.3 ml) were kept consistent. We acknowledge the importance of your point regarding the bacterial load in whole lung tissue as a reflection of overall infection severity.

To investigate the role of caspase-1 in murine pulmonary *E. coli* infection, we conducted an *in vivo* experiment where the only controlled variable was the presence or absence of caspase-1 in the mice. We instilled the same amount of bacteria into *Casp1*$^{-/-}$ mice and their littermate WT controls. Caspase-1 knockout resulted in better bacterial clearance and improved survival. While we agree that bacterial load and inflammation and effects of IL-1β, respiration, temperature, neutrophil number, and

neutrophil necroptosis can all influence survival and injury to some extent, these outcomes are also resulted from caspase-1 knockout. We think that the bacteria load and immune cells are indeed very important determinants in mice survival and that the protective effect of caspase-1 knockout may be mainly from them. We have include this in our discussion in line 430-434: "bacterial load and inflammation are critical factors that can influence mouse survival during disease. Albeit caspase-1 modulates bacterial clearance and inflammation status, it remains unclear how bacterial load, inflammation, and neutrophil necroptosis individually contribute to mouse survival."

3. The authors report that IL-1 is not involved because anakinra could not rescue the mice from lethal pneumonia (line 275). However, the authors have not shown that anakinra as delivered was available in the lung, nor that it inhibited IL-1 signaling like claimed. Actual signaling of IL-1 in the lung should to be measured to know whether the drug is having the stated effect, and this mechanism needs to be confirmed using IL-1R KO mice.

Response: We appreciated this good suggestion. We administered anakinra to mice both intratracheally and intraperitoneally at a previously published and validated dose. In a previous study, *Ccr2*$^{-/-}$ mice infected with *K. pneumoniae* showed improved survival when treated with 200 μg/kg anakinra intratracheally (17) (Ref fig. 1). Another study also showed that intraperitoneally treated with 100mg/kg anakinra effectively downregulated lung IL-1β in *P. aeruginosa* infected mice (18) (Ref fig. 2). However, we did not have access to IL-1R knockout mice for this experiment. To further support

our conclusions, we additionally treated mice intraperitoneally with an anti-mIL-1β antibody at a widely used dose (200 μg each) (19-22) for IL-1β neutralization. Our findings indicated that neutralization of IL-1β did not attenuate *E.coli* pneumonia in the mice (Fig. S4F). Thank you for your consideration of these results.

Ref fig. 1

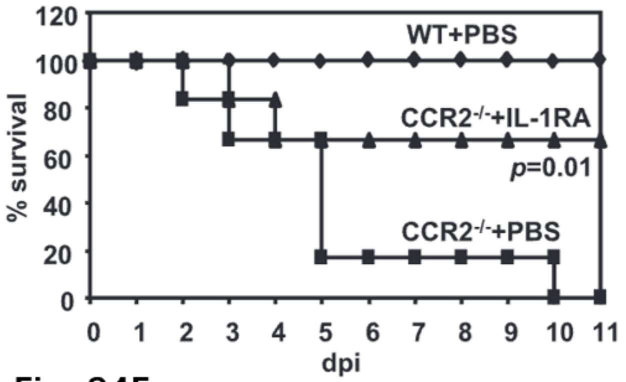

Ref fig. 2

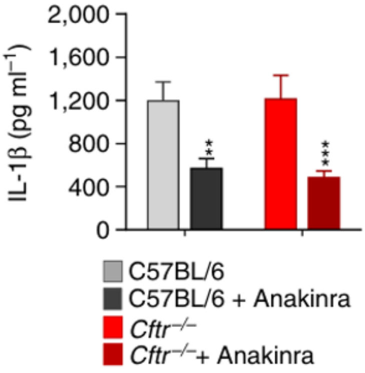

Fig. S4F

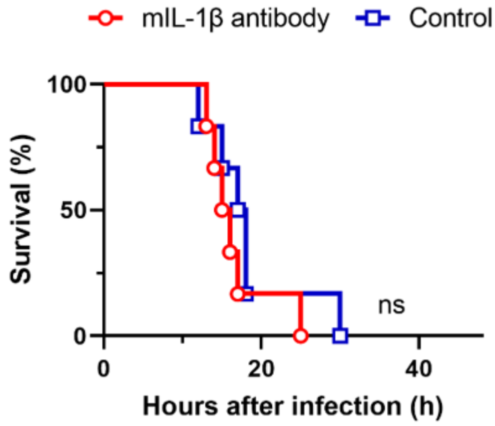

The effect of mice IL-1β blockade on survival of mice *E.coli* lung infection.

(Ref fig. 1) Alveolar deposition of IL-1ra attenuates epithelial injury without affecting host defense in Klebsiella pneumoniae pneumonia. Survival rates of WT and *Ccr2*$^{-/-}$ mice intratracheally treated with 10μg IL-1ra or PBS at the time point of infection with 10^5 *K. pneumoniae* (17).

(Ref fig. 2) C57BL/6 and *Cftr*$^{-/-}$ mice (n=6) were infected intranasally with live *P.*

aeruginosa and treated intraperitoneally with anakinra (100 mg kg^{-1}). IL-1β levels in lung homogenates of anakinra-treated mice (18).

(Fig. S4F) WT mice were pretreated intratracheally with mIL-1β antibody/control antibody and subjected to lung *E.coli* infection. Survival was monitored for 48 hours. Statistical differences were determined by Mantel–Cox test. ns, not significant.

4. Similarly, the authors need to show that the cell ablations of Fig 1F and 1G are leading to actual depletion of these cells in the experiment.

Response: Yes, thank you for your comment. To address your concern, we showed the neutrophil depletion efficiency analysis as following figure. The macrophage depletion method utilized in our study is based on a previously published and validated approach from our group (9).

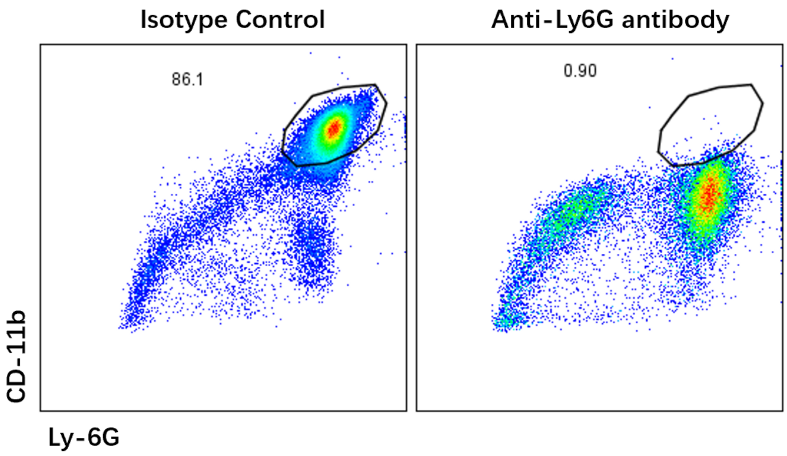

The verification of neutrophil depletion.

Mice were intraperitoneally injected with 200 μg anti-ly6G antibody or isotype control 2 days before the experiment and were intratracheally injected with 100 μg anti-ly6G antibody or isotype control on the day of the experiment. Depletion efficiency was

confirmed by calculating neutrophils ($CD11b^{+}Ly6G^{+}$%) with flow cytometry. This result is a representative of 2 independent experiments.

5. The experiments of Fig 5F and 5G are an unconvincing demonstration of PolyIC uptake. F shows association with cells, which could be sticking on the surface and not uptake. G is very low magnification, and appears to show staining of the whole cell for some cells, and no staining at all for others, which isn't a demonstration of colocalization. Higher-magnification imaging, with membrane markers if needed, should be used to show polyIC is intracellular, and colocalization requires that there are areas that there are areas lacking both polyIC and ZBP1, not just the whole cell imaged as positive for both.

Response: Yes, thank you for the good suggestion. We have utilized higher magnification imaging, which demonstrates the colocalization of poly(I:C) and ZBP1 signals in bacterial supernatant treated purified neutrophils (Fig. 5G).

Fig. 5G

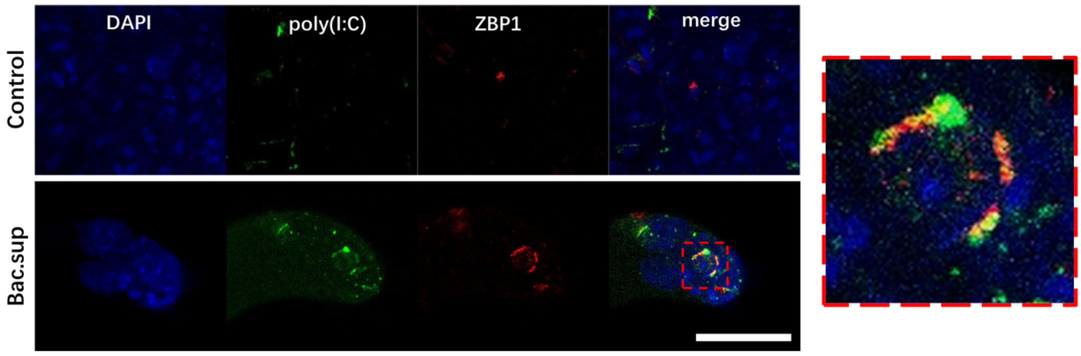

(Fig. 5G) Immunofluorescence of neutrophils cocultured with control/Bac.sup and FITC-conjugated poly(I:C) using anti-ZBP1 antibody. Scale bars, 25 μm.

6. Figure 2E compares proteins in BALF cells, which are mostly neutrophils, to the tissue, which is mostly not. For one, the methods for this experiment are unclear, not in the text, the legend, or figure is it stated how and when these samples were derived. I will assume this is just an omission and that they were infected mice, though not stated, and for a similar amount of time as other experiments. However, there are several biases in these comparisons needing controls including uninfected mice and purified neutrophils, and a survivorship bias, as their other data show evidence of significant death by this point, so it is living cells that have p-MLKL, arguing that the dead cells might have died by other means. This major point also needs the good blotting control using the *MLKL*$^{-/-}$ mice of Fig 3G

Response: Yes. We apologize for any confusion caused by the unclear methods in this experiment, and we have added a section in the Materials/Subjects and Methods to provide a detailed description of the procedure at line 511-520:

"**Collection of BALF Cells and Lung Samples for Western Blot Analysis**

Mice were sacrificed 12 hours post *E.coli* infection. Bronchoalveolar lavage fluid (BALF) was collected by performing three washes with 0.5 ml of PBS, followed by centrifugation at 800 g for 5 minutes to isolate BALF cells. The lungs were subsequently harvested and stored in liquid nitrogen. For protein extraction, RIPA buffer was added to both the BALF cells and the lung tissue. The samples were then subjected to ultrasonic fragmentation at 4 °C, 50 W for 3 minutes. Following this, the samples were centrifuged at 12,000 rpm for 10 minutes, and the supernatants were collected for protein concentration analysis."

Based on the results shown in Figure 1A, the mice did not begin to show signs of mortality at this time point, which is why we chose 12 hours as our sample collection time.

Additionally, we included uninfected mice, purified neutrophils, and MLKL knockout mice as negative controls in our Western blot analysis (Fig. 2D).

You raised a point regarding the presence of p-MLKL in living cells, and the possibility that dead cells might have died through other means. In response to specific stimuli, cells can generate p-MLKL, leading to membrane permeabilization and ultimately lytic cell death. Therefore, if the cells we collected—including membrane-intact living cells and membrane-permeabilized dying cells—are undergoing such continent process and expressing p-MLKL, we believe it is reasonable to conclude that p-MLKL related necroptosis may be a contributing factor to the observed cell death.

Fig. 2D

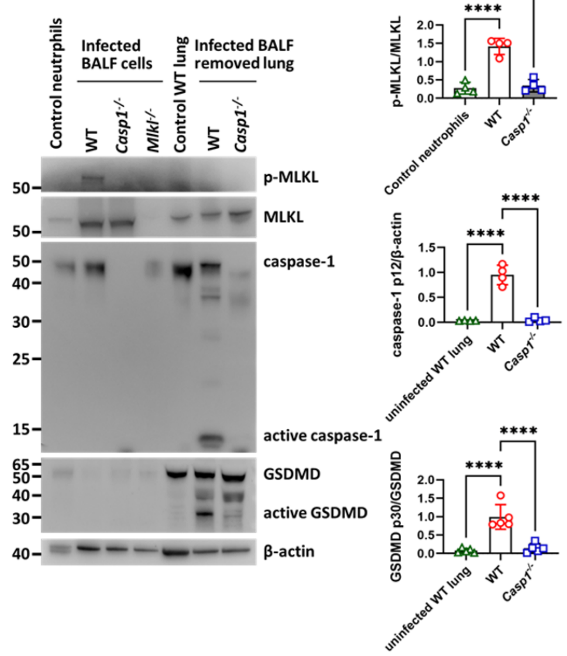

Fig. 2D. The effect of caspase-1 on necroptosis and pyroptosis of BALF cells and BALF

removed lung in *E.coli* infected mice.

(Fig. 2D) BALF cells (containing > 95% neutrophils) and lung tissue without BALF cells from indicated mice were collected and immunoblotted for programmed cell death-related proteins and grey mean values for bands were calculated and plotted.

Data are shown as the mean ± SD. Statistical differences were determined by one-way ANOVA. ****P < 0.0001.

7. 3E requires PBS control for infection-independent effects of the drug on neutrophil death

Response: Yes. Thank you for the good suggestion. We have added the PBS control for measuring infection-independent effect of the drug. Thank you for your attention on this result.

Fig. 3E

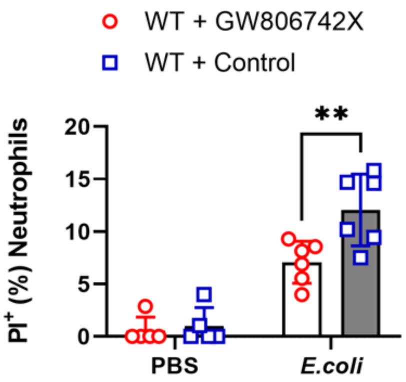

(Fig. 3E) BALF from WT mice treated with the MLKL inhibitor GW806742X or control solvent was harvested at 12 hours after intratracheal PBS instillation (n = 5 each) or pulmonary *E. coli* infection (n = 6 each), and PI^+ neutrophil proportion were determined.

Data are shown as the mean ± SD. Statistical differences were determined by one-way

ANOVA. **P < 0.01.

8. The cytometry of Fig 4A 4B requires additional details on markers, compensation, and gating, total and live/dead cells of each population, and controls of uninfected mice. As it is, it is not clear how abundant the cell populations are, how accurate their identification is, and how reliable the FLICA staining is. In particular, the cell processing method is quite harsh and expected to lead to significant cell death, which can bias these populations, and give false positives/negatives.

Response: Thank you for the good suggestion. We performed the gating strategy (Fig. S4A) and included controlled uninfected mice (Fig. 4A). Since there was also some cell death events in the control uninfected mice due to the processing method, we only focus on caspase-1 activate cells (FLICA$^+$ cells).

Fig. S4A

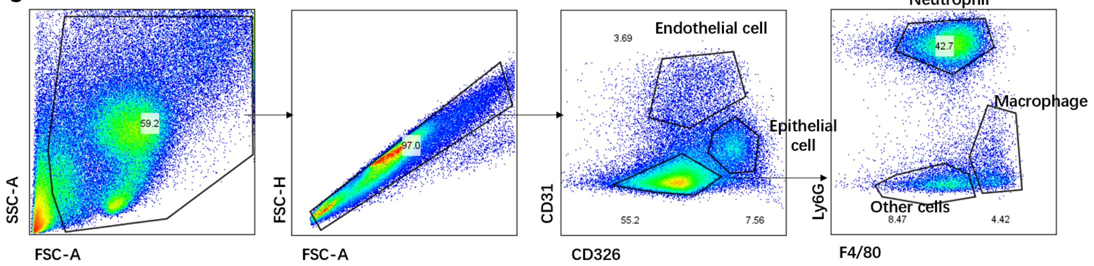

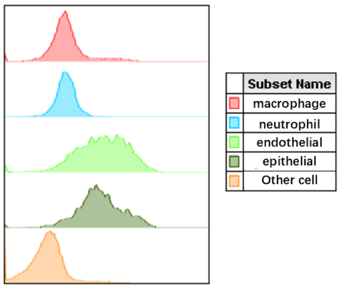

Fig. 4A

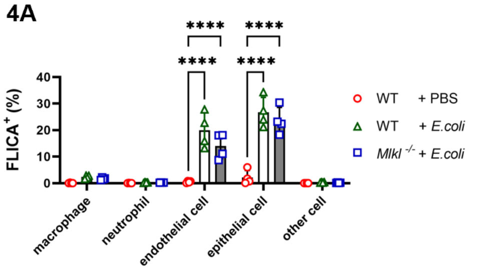

(Fig. S4A) Gating strategy of flow cytometry analysis.

(Fig. 4A) The proportion of neutrophils, macrophages, endothelial cells, epithelial cells and other cells with activated caspase-1 in the lungs of WT, *Casp1*$^{-/-}$ and *Mlkl*$^{-/-}$ mice at

12 hours after pulmonary *E.coli* infection was assessed by FLICA staining using flow cytometry (n = 4).

Data are shown as the mean ± SD. Statistical differences were determined by one-way ANOVA. ****P < 0.0001.

9. A statistician should review the methods, which are applied inconsistently between figures. For example, two-way ANOVA with Tukey-Kramer's post hoc analysis is done for Fig 3 (C, D, E and F), but some of these are pairwise, others multivariate, and methods are applied inconsistently between different figures of similar data.

Response: Thank you for the comments. We apologized for these mistakes in statistical analysis. We have checked all of the data with a statistician in the revised manuscript.

10. There are gaps in the claims on the specificity of each cell death & sensing mechanism to each cell type. This is most cleanly and directly addressed by lineage-specific knockout. It minimum, these limitations should be discussed and redundancies between these pathways should be excluded including the effect of dsRNA antibody on neutrophil death in infected *Casp1*$^{-/-}$ and *Mlkl*$^{-/-}$ mice, the effect of *Mlkl*$^{-/-}$ on FLICA cells, and histological examinations added to the major mechanistic points (not just Fig 1B)

Response: Thank you very much for the suggestions. We acknowledge that our previous conclusion was too strong based on the experiments conducted. As a supplement, we have revised the overall claim to "non-hematopoietic caspase-1-drives

neutrophil necroptosis and dampen host defense against bacterial pneumonia." Additionally, we generated a bone marrow transplant mouse model for this experiment. The results indicate that non-hematopoietic caspase-1 or GSDMD knockout protected mice, leading to improved survival, reduced neutrophil death and reduced dsRNA release (Fig. 4, H and I; Fig. 5E).

Fig. 4H

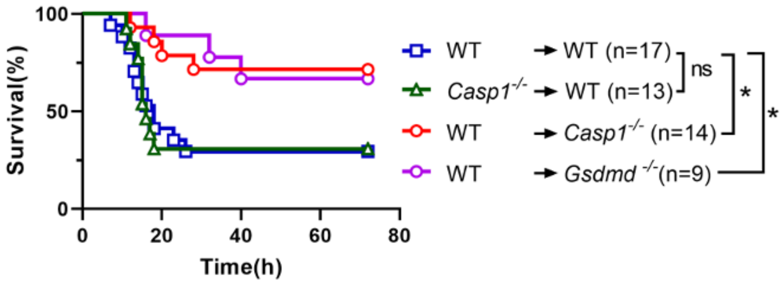

Fig. 4I

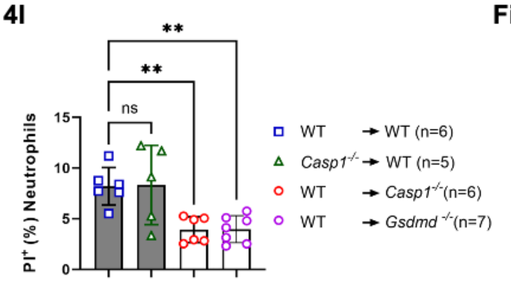

Fig. 5E

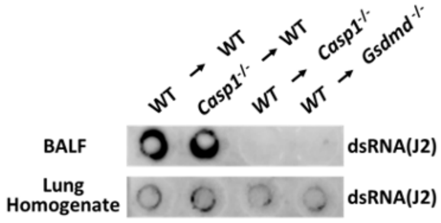

Reciprocal bone marrow chimera experiments.

(Fig. 4, H and I) Chimeric mice were created by reciprocal bone marrow transplantation between WT, *Casp1*$^{-/-}$ and *Gsdmd*$^{-/-}$ mice as indicated, and then mice were subjected to *E.coli*-induced pneumonia. Mouse survival was monitored (H). BALF cells were collected at 12 hours and neutrophil death was determined by PI staining and flow cytometry (I). The survival experiment was conducted across three independent replicates, with each replicate including 3–6 mice.

(Fig. 5E) Dot blot of BALF from indicated chimeric mice at 12 hours after pulmonary *E.coli* infection using anti-dsRNA antibody.

Data are shown as the mean ± SD. Statistical differences were determined by one-way ANOVA (4I) and Mantel–Cox test (4H). *P < 0.05; **P < 0.01. ns, not significant.

Furthermore, we examined the effect of dsRNA antibody on neutrophil death in infected *Casp1*$^{-/-}$ and *Mlkl*$^{-/-}$mice. The results showed that treatment with anti-dsRNA antibody did not reduce neutrophil death in *Casp1* or *Mlkl* knockout mice (Fig. 5, L and M).

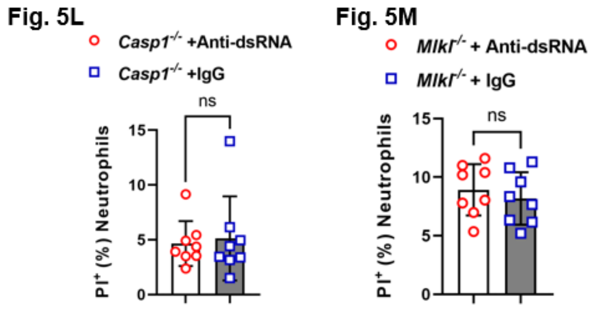

Treatment with anti-dsRNA antibody did not reduce neutrophil death of *Casp1*$^{-/-}$ and *Mlkl*$^{-/-}$ mice.

(Fig. 5, L and M) Proportion of PI+ neutrophils in the BALF of *Casp1*$^{-/-}$ (L) and *Mlkl*$^{-/-}$ (M) mice treated with anti-dsRNA antibody or IgG control antibody (n = 8 each) at 12 h after pulmonary *E.coli* infection.

Data are shown as the means ± SD. Statistical differences were determined by the Student's *t* test. ns, not significant.

We also assessed the impact of *Mlkl*$^{-/-}$ on FLICA cells, the result showed that caspase-1 was activated in epithelial and endothelial cells of *Mlkl*$^{-/-}$ mice (Fig. 4A).

Fig. 4A

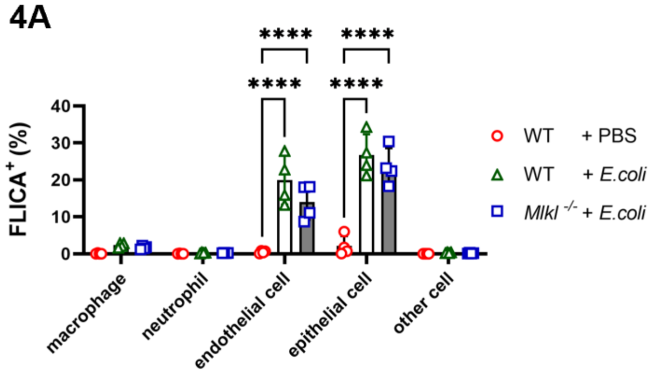

(Fig. 4A) The proportion of neutrophils, macrophages, endothelial cells, epithelial cells and other cells with activated caspase-1 in the lungs of WT, *Casp1*$^{-/-}$ and *Mlkl*$^{-/-}$ mice at 12 hours after pulmonary *E.coli* infection was assessed by FLICA staining using flow cytometry (n = 4).

Data are shown as the mean ± SD. Statistical differences were determined by one-way ANOVA. ****P < 0.0001.

Moreover, we added histological examinations to each major mechanistic point (Fig. S3D and Fig. S6, D and E).

Fig. S3D Fig. S6D Fig. S6E

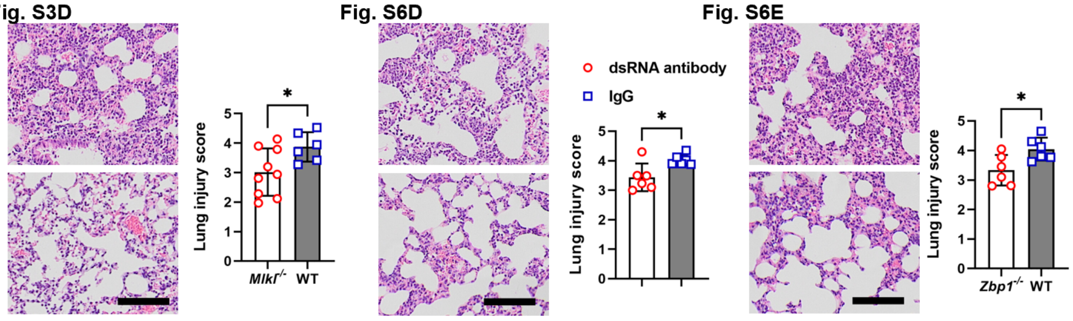

Histological examinations to each major mechanistic points including MLKL and ZBP1 knockout and dsRNA treatment.

(Fig. S3D) Representative images of hematoxylin and eosin staining of the lungs from *Mlkl*$^{-/-}$ mice (n = 9) and WT littermate mice (n = 6) at 12 hours after *E.coli* infection

and quantification of lung injury scores. Scale bars, 100 μm.

(Fig. S6D) Representative images of hematoxylin and eosin staining of the lungs from *Zbp1*$^{-/-}$ mice and WT littermate mice (n = 6 each) at 12 hours after *E.coli* infection and quantification of lung injury scores. Scale bars, 100 μm.

(Fig. S6E) Representative images of hematoxylin and eosin staining of the lungs from anti-dsRNA treated WT mice and Control IgG antibody treated cohoused WT mice (n = 6 each) at 12 hours after *E.coli* infection and quantification of lung injury scores. Scale bars, 100 μm.

Data are shown as the mean ± SD. Statistical differences were determined by Student's *t* test. *P < 0.05;

Reviewer #3:

1. The presented data did not directly demonstrate in vivo that pyroptotic epithelial cells are responsible for dsRNA release in BALF. It is also not clear which cell death (Pyroptosis versus necroptosis) occurs first, or whether dead neutrophil causes epithelial cell damage and release of dsRNA. To address these, the authors should use epithelial cell-specific Caspase-1 deficient mice. However, generating such a mouse line will take at least 6 months. Instead, adoptive transfer experiment could provide some support to the conclusions. Bone marrow-derived neutrophils from wild-type mice could be adoptively transferred to UV-irradiated Caspase-1 KO mice and test pneumonia outcomes (mice survival; bacterial and immune cell counts; dsRNA level in the BALF and lungs; neutrophil and epithelial cell death and so on). The mice should be protected if the proposed conclusion is correct (i.e., pyroptotic epithelial cells released dsRNA to induce necroptotic neutrophils). Also, bone marrow-derived neutrophils from Caspase-1 KO mice could be adoptively transferred to UV-irradiated wild-type mice and test pneumonia outcomes. The experiment outcome could assess whether epithelial cell death or neutrophil death occurs first.

Response: Yes, thank you for the good suggestion. According to your suggestion, we have adoptively transplanted wild-type (WT) bone marrow cells into WT, *Casp1*$^{-/-}$, and *Gsdmd*$^{-/-}$ mice. The results demonstrated that *Casp1*$^{-/-}$ and *Gsdmd*$^{-/-}$ mice receiving WT bone marrow cells exhibited a lower neutrophil death rate, lower dsRNA release and were better protected from *E.coli* pneumonia (Fig. 4, H and I and Fig. 5E).

Fig. 4H

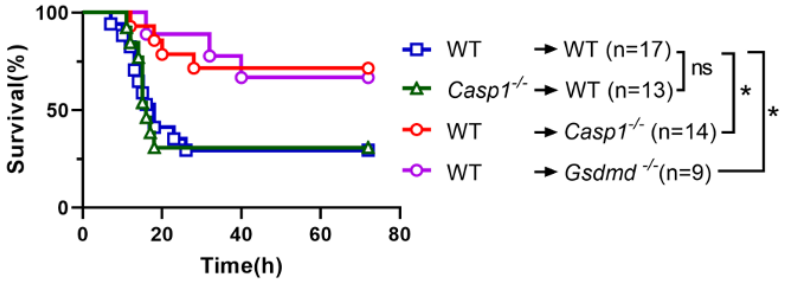

Fig. 4I

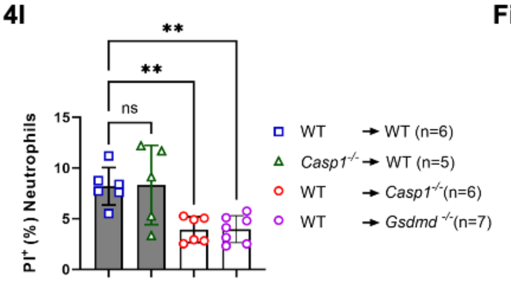

Fig. 5E

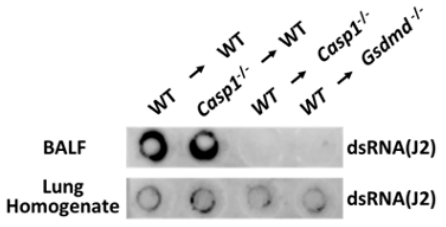

Reciprocal bone marrow chimera experiments.

(Fig. 4, H and I) Chimeric mice were created by reciprocal bone marrow transplantation between WT, *Casp1*$^{-/-}$ and *Gsdmd*$^{-/-}$ mice as indicated, and then mice were subjected to *E.coli*-induced pneumonia. Mouse survival was monitored (H). BALF cells were collected at 12 hours and neutrophil death was determined by PI staining and flow cytometry (I). The survival experiment was conducted across three independent replicates, with each replicate including 3–6 mice.

(Fig. 5E) Dot blot of BALF from indicated chimeric mice at 12 hours after pulmonary *E.coli* infection using anti-dsRNA antibody.

Data are shown as the mean ± SD. Statistical differences were determined by one-way ANOVA (4I) and Mantel–Cox test (4H). *P < 0.05; **P < 0.01. ns, not significant.

Additionally, we found that the lungs of *Mlkl*$^{-/-}$ mice displayed active caspase-1 signaling, further indicating that pyroptosis occurs upstream of neutrophil necroptosis

(Fig. 4A).

Fig. 4A

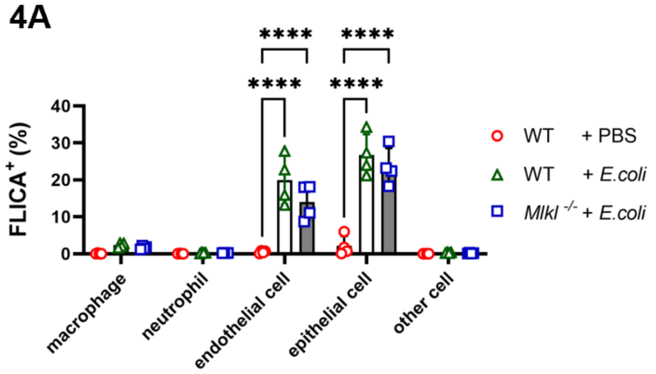

(Fig. 4A) The proportion of neutrophils, macrophages, endothelial cells, epithelial cells and other cells with activated caspase-1 in the lungs of WT, *Casp1*$^{-/-}$ and *Mlkl*$^{-/-}$ mice at 12 hours after pulmonary *E.coli* infection was assessed by FLICA staining using flow cytometry (n = 4).

Data are shown as the mean ± SD. Statistical differences were determined by one-way ANOVA. ****P < 0.0001.

2. Which type of epithelial cells are affected? the authors should combine Tunel staining of histology sections from wild-type and Caspase1 KO with different cell markers including type I epithelial, type II epithelial, macrophages, neutrophils, and monocytes.

Response: Yes, thank you very much for the suggestion. We performed TUNEL staining with various cell markers, including the epithelial marker CD326, endothelial marker CD31, type II epithelial marker SPC, macrophage marker F4/80, neutrophil marker Ly-6G, and monocyte marker CD14, on lung sections from WT and *Casp1*$^{-/-}$ mice. Our findings indicated that both WT and *Casp1*$^{-/-}$ mice exhibited comparable TUNEL signals in macrophages (F4/80^{+}), with minimal signals observed in monocytes

(CD14$^+$) and type II epithelial cells (SPC$^+$). Notably, WT mice showed significantly higher TUNEL signals in epithelial cells (CD326$^+$), endothelial cells (CD31$^+$), and neutrophils (Ly6G$^+$) in the following figure. These results suggest that type I epithelial cells, endothelial cells, and neutrophils are the primarily affected cell types in WT mice compared to *Casp1*$^{-/-}$ mice.

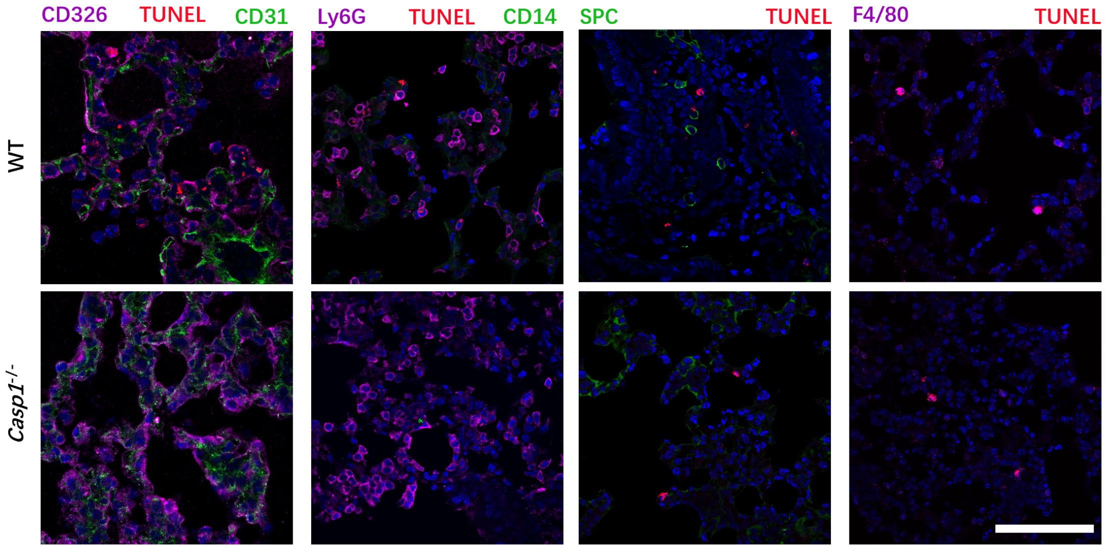

TUNEL staining of histology sections with different cell markers from WT and *Casp1*$^{-/-}$ *E.coli* infected mice.

WT and *Casp1*$^{-/-}$ mice lung were harvest at 12 hours post *E.coli* infection. TUNEL staining of lung sections with different cell markers as indicated was performed. Data are representative of 3 independent experiments.

3. A combination of LPS + poly I:C stimulation to induce necroptotic neutrophils in vitro and in vivo should be tested. This will provide evidence that dsRNA from dying cells along with LPS from bacteria are sufficient to derive necroptotic neutrophils.

Response: Yes, this is a good suggestion. As you suggested, we treated purified

neutrophils with LPS (1μg/ml) and poly(I:C) (1μg/ml). The result indicated that this combination could trigger neutrophil death (Left figure). However, treatment with poly(I:C) in combination with bacterial supernatant resulted in significant higher neutrophil death (Right figure). Thus, we thought that besides LPS there were some other components that facilitate the dsRNA related cell death under such circumstances.

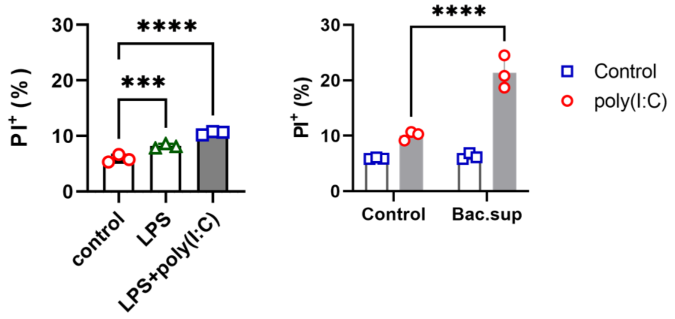

The ability of poly(I:C) to induce neutrophil death with the combination of LPS or bacteria supernatant.

(Left) Purified WT neutrophils were stimulated with LPS (1μg/ml) alone or in combination of poly(I:C) for 4 hours in 37°C and cell death was monitored by PI staining and flow cytometry (n=3).

(Right) neutrophil death (PI+) were measured by flow cytometry after stimulation with *E.coli* culture supernatant (Bac. Sup) or control medium with/without FITC-conjugated poly(I:C) (n = 3).

Data are shown as the means ± SD. Statistical differences were determined by one-way ANOVA. ***P < 0.001; ****P < 0.0001.

4. The authors need to measure bacterial loads in the lungs. Measuring bacterial counts in the BALF is not sufficient to conclude bacterial clearance. Bacterial counts in BALF

depend on the efficiency of performing the lavage (do the authors use equal volume to lavage mice and whether end up with equal volumes of BALF from wild type and KOs. Also, immune cell counts in the lungs are a better predictor of disease outcomes than BALF.

Response: Thank you for the comments. We agree that bacterial counts and immune cell assessments in the whole lung are important predictors of disease outcome. However, bacterial counts from bronchoalveolar lavage fluid (BALF) are widely used and validated as a reliable method to measure airway bacterial clearance (14-16). Based on our methods, the volume of PBS injected into each mouse (0.5 ml × 3, totaling 1.5 ml) and the volume extracted (1.2–1.3 ml) were kept consistent. We acknowledge the importance of your point regarding the bacterial load in whole lung tissue as a reflection of overall infection severity. We will consider this suggestion in future studies.

5. Fig. 1D showed a massive loss of macrophage during infection. Are they dying? If so, which cell deaths are responsible, and could they contribute to dsRNA release? Why there are lower macrophage counts in uninfected wild-type relative to Caspase-1 KO? Do Caspase-1 KO mice experience some sort of low-grade inflammation, especially in the lungs?

Response: Yes, thank you for the comment. We observed a significant decrease in macrophages in both WT and *Casp1*$^{-/-}$ mice. Coupled with the results from TUNEL staining as shown in following figure, it appears that macrophages from both groups are experiencing similar rates of cell death. To further investigate the influence of

macrophages, we utilized chloride liposomes to deplete macrophages in *Casp1*$^{-/-}$ mice and found that their survival rate was not significantly affected (Fig. 1G). This suggests that macrophages have a minimal impact on the pathogenesis of *E.coli* airway infection.

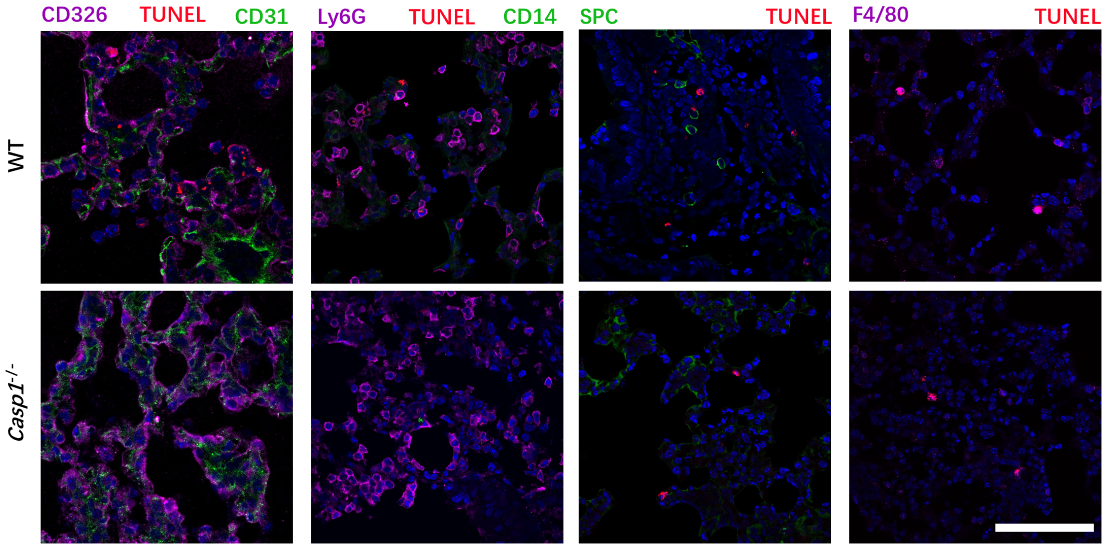

TUNEL staining of histology sections with different cell markers from WT and *Casp1*$^{-/-}$ *E.coli* infected mice.

WT and *Casp1*$^{-/-}$ mice lung were harvest at 12 hours post *E.coli* infection. TUNEL staining of lung sections with different cell markers as indicated was performed. Data are representative of 3 independent experiments.

According to our findings in following figure as shown in following figure, there is nearly none dsRNA released from *Casp1*$^{-/-}$ mice. Thus, we thought that the contribution of dying macrophages to BALF dsRNA is minimal.

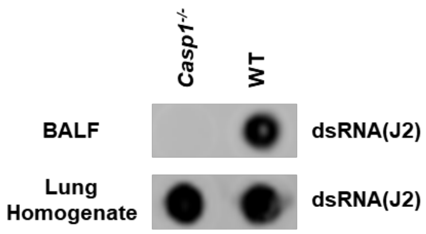

dsRNA released from BALF of *E.coli* infected WT or *Casp1*$^{-/-}$ mice.

Dot blot of dsRNA from BALF of WT or *Casp1*$^{-/-}$ mice 12 hours after *E.coli* lung infection. Data are representatives of 3 independent experiments.

6. All immune cells count, and bacterial burden should be graphed using a log y-axis scale. This way we could see the lower number of immune cells and bacteria that are present in some of the conditions.

Response: Thank you for your suggestion. We are sorry for any inconvenience caused by unprecise graphical structure. We have adjusted the immune cell counts and bacterial burden to a logarithmic y-axis scale, which allows for better visualization of the lower numbers in the graph.

7. It is not clear whether BALF cells in Fig. 2C or Fig. 4H that induced necroptosis are macrophages, monocytes, or neutrophils. Although neutrophil counts are higher in BALF, the amount of protein in these cells is very low compared to macrophages and monocytes. The authors should either sort these cells or magnetic purify them. Similarly, the remnant lung tissue after lavage is not only epithelial cells. Many other cells including interstitial macrophages could be the driver for activation of Caspase-1 and GSDMD.

Response: Yes, this is a good suggestion. Based on the results of our flow cytometry analysis, neutrophils comprise more than 95% of the cell count in the BALF of infected mice. To further address your question, we sorted neutrophils and measured p-MLKL levels using flow cytometry (Fig. 2E).

Fig. 2E

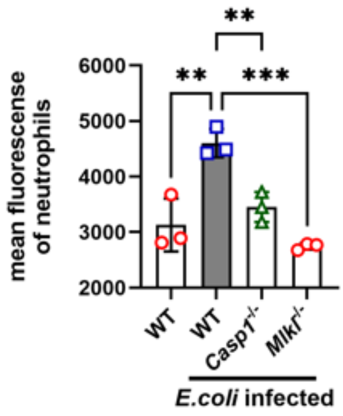

The effect of caspase-1 knockout on p-MLKL fluorescence of infected BALF neutrophils.

(Fig. 2E) BALF neutrophils from indicated mice were harvested at 12 hours after *E.coli* lung infection and stained with p-MLKL antibody and mean fluorescence was measured by flow cytometry.

Data are shown as the mean ± SD. Statistical differences were determined by one-way ANOVA. $^{**}P < 0.01$; $^{***}P < 0.001$.

Additionally, we also assessed active caspase-1 in non-hematopoietic cells through flow cytometry (Fig. 4A). The result showed that epithelial cells and endothelial cells have more activate caspase-1 than other hematopoietic cells, and epithelial cells had more activate caspase-1 than endothelial cells.

Fig. 4A

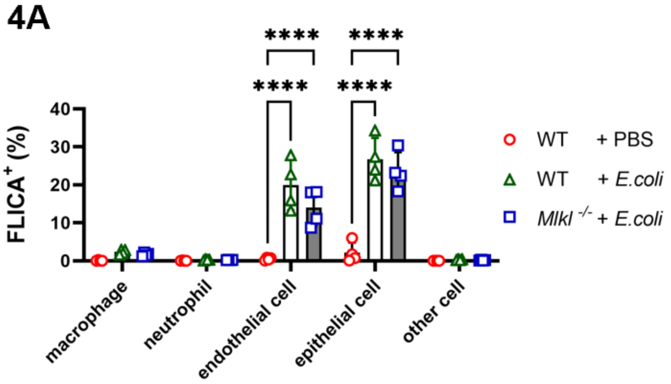

(Fig. 4A) The proportion of neutrophils, macrophages, endothelial cells, epithelial cells and other cells with activated caspase-1 in the lungs of WT, *Casp1*$^{-/-}$ and *Mlkl*$^{-/-}$ mice at 12 hours after pulmonary *E.coli* infection was assessed by FLICA staining using flow cytometry (n = 4).

Data are shown as the mean ± SD. Statistical differences were determined by one-way ANOVA. ****P < 0.0001.

8. Treated mice with control solvents such as DMSO, Saline, control IgG…etc are dying at a faster rate than untreated mice. Examples include in fig. 3A, fig. 3B, fig.5I, supplementary fig. 4D and 4E. Do the authors know why this is the case? Does DMSO or saline treatment make the mice more susceptible to infection? This should be emphasized.

Response: Yes, thank you for the comments. The wild-type (WT) mice treated with DMSO, saline, or control IgG were directly purchased from an animal company. In contrast, the control WT mice in the experiments with *Casp1*$^{-/-}$, *Mlkl*$^{-/-}$, and *Zbp1*$^{-/-}$ mice, are littermates and are all offspring of *Casp1*$^{+/-}$, *Mlkl*$^{+/-}$, and *Zbp1*$^{+/-}$ mice that we raised ourselves in our university facilities.

We acknowledge that there may be differences in breeding and living conditions that could contribute to variations in survival rates. However, each knockout mouse experiment is controlled using the corresponding littermates, and the animals purchased from the company are controlled with their own cohoused groups.

Minor issues:

Reviewer #3:

1. Impact neutrophil depletion on wild-type mice should be provided. Does neutrophil depletion impact bacterial clearance in wild-type mice?

Response: Yes. According to your suggestion, we assessed the impact of neutrophil depletion on WT mice. The neutrophil-depleted mice began to exhibit signs of mortality at an earlier time point (6–7 hours post-infection), necessitating the collection of BALF samples at 5 hours after *E.coli* instillation. The results indicated that neutrophil depletion did not significantly affect bacterial clearance in wild-type mice at least in early time point as shown in following figure.

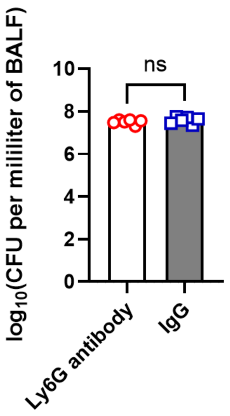

The effect of neutrophil depletion on bacterial clearance in wild-type mice.

BALF were collected from Ly6G antibody or control IgG antibody treated mice 12

hours after *E.coli* lung infection and bacteria burden was determined and expressed as CFU/ml.

Data are shown as the mean ± SD. Statistical differences were determined by Student's *t* test. ns, not significant.

2. The lung injury score graph (Fig. 1B) should be fixed back to a standard y-axis graph. It is not clear why a two-segment y-axis was used.

Response: Yes, thank you for your suggestions. We have changed the lung injury score graph into a standard y-axis graph.

3. The last section of the results was poorly written (lines 325-381). There are a lot of missing spaces, the authors used "we next" several times to start a new sentence. It seems that the authors listing experiment findings without rationale or context.

Response: We appreciate your kind suggestion and have rewritten this section accordingly.

4. In line 41 "conbined" should be fixed to combined.

Response: We are sorry for the mistake. We have corrected the misspelled word.

5. The authors should immunofluorescence stain other BALF and lung cells with pMLKL antibody, like supplement fig. 2H. This could provide evidence that necroptosis impacted only neutrophils and/or other cells.

Response: Yes, thank you for the suggestion. We stained for p-MLKL in *E.coli*-infected lung sections, and p-MLKL was found to localize exclusively with neutrophils, identified as Ly-6G positive.

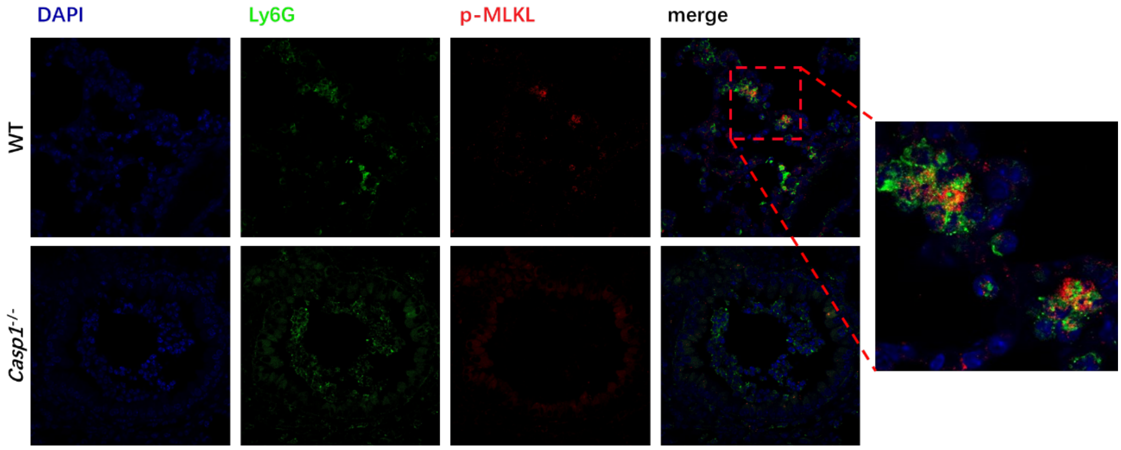

The p-MLKL staining of lung section with neutrophils.

WT and *Casp1*$^{-/-}$ mice lung were harvest at 12 hours post *E.coli* infection. p-MLKL staining of lung sections with neutrophil markers was performed by immunofluorescence. Data are representative of 3 independent experiments.

6. Does pyroptosis still occur in epithelial cells when the mice were treated with GW806742X or in Mlkl KO mice?

Response: Yes, thank you for the suggestion. We stained *Mlkl*$^{-/-}$ mice with FLICA and observed that epithelial and endothelial cells still exhibited activated caspase-1 (Fig. 4A). This result indicates that pyroptosis still occurs in epithelial cells in *Mlkl*$^{-/-}$ mice.

Fig. 4A

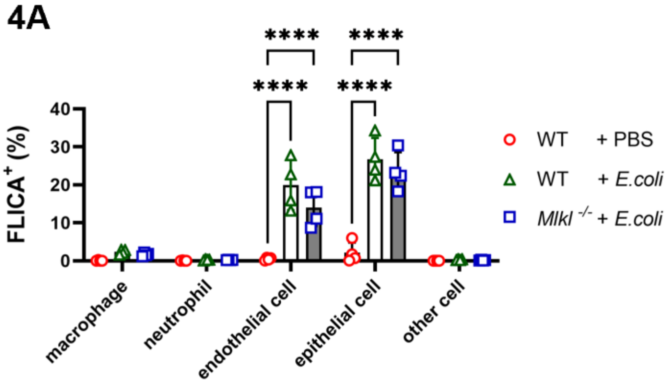

(Fig. 4A) The proportion of neutrophils, macrophages, endothelial cells, epithelial cells and other cells with activated caspase-1 in the lungs of WT, *Casp1*$^{-/-}$ and *Mlkl*$^{-/-}$ mice at 12 hours after pulmonary *E.coli* infection was assessed by FLICA staining using flow cytometry (n = 4).

Data are shown as the mean ± SD. Statistical differences were determined by one-way ANOVA. ****P < 0.0001.

7. It is not clear how purified neutrophils are dying in response to *E.coli* and heat-killed *E.coli* in supplementary fig. 4A. The authors need to include more detail on how infections were performed. What is the multiplicity of infection (MOI) used and what time point post-infection cell death was analyzed? Are extracellular bacteria washed or kept in the media? Also, LPS-treated purified neutrophils induce activation of MLKL but no cell death. The authors need to explain why LPS stimulation is insufficient to trigger necroptosis.

Response: Yes, thank you for the suggestion. We have included detailed methods in the methods section in line 630-637:

***In vitro* stimulation and cell death analysis of neutrophils**

E.coli (MOI = 2), LPS (1μg/ml), Heated *E.coli* (MOI = 20) or collected supernatants were added to the neutrophils 1 hour after inhibitor (GSK-872, GW806742X, NEC-1 or Z-VAD FMK) treatment. Four hours after stimulation, the cells were centrifuged and lysates were collected for western blot analysis. In some experiments, the nuclei were stained with 2.5 μg/ml PI, and data were collected using a Beckman Coulter DxFLEX flow cytometer (Beckman Coulter Life Sciences) and analyzed with FlowJo v.10 software.

As shown in Fig. S4A, LPS-stimulated neutrophils actually exhibit a higher percentage of death compared to PBS-treated neutrophils. We thought that LPS has triggered very mild neutrophil necroptosis. However, the *in vivo* BALF cell samples from *Casp1*$^{-/-}$ mice showed no p-MLKL activation (Fig. 2D). Our *in vivo* model suggested that neutrophil necroptosis is not directly induced by bacteria stimulation. Bacteria first influence the non-hematopoietic cells and then the non-hematopoietic released matter contribute to neutrophil necroptosis with bacteria supernatant. We think that LPS alone may not be sufficient to induce enough neutrophil necroptosis. It is possible that LPS with other substances in bacteria supernatant and host cells may collaborate to further promote neutrophil death. We have included this in our discussion in line 410-413: "LPS from gram-negative bacteria can induce cell death. Our results showed that LPS induced neutrophil p-MLKL but only very mild cell death. It is possible that LPS with other substances in bacteria supernatant and host cells may collaborate to further promote neutrophil death."

Fig. S4A

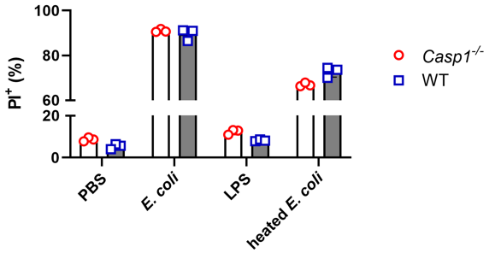

Fig. 2D

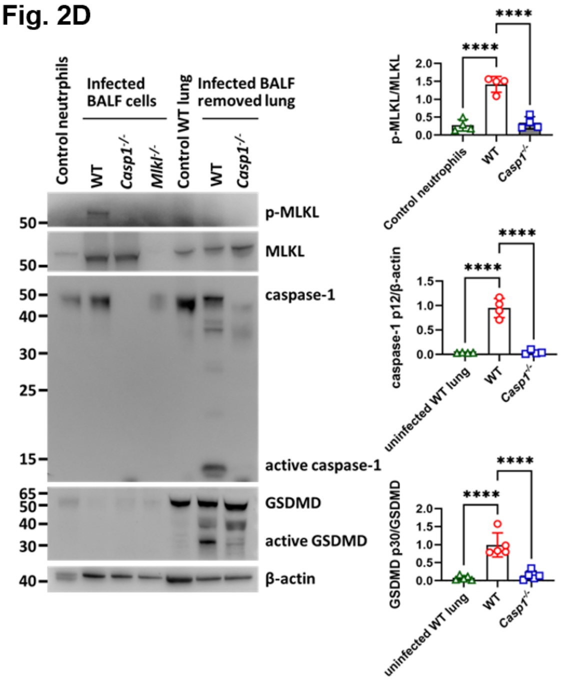

(Fig. S4A) The proportion of PI+ neutrophils from *Casp1-/-* and WT mice after stimulation with LPS, live *E. coli* and heat-inactivated *E. coli* and PBS (n = 3).

(Fig. 2D) BALF cells (containing > 95% neutrophils) and lung tissue without BALF cells from indicated mice were collected and immunoblotted for programmed cell death-related proteins and grey mean values for bands were calculated and plotted. Data are shown as the mean ± SD. Statistical differences were determined by one-way ANOVA. ****P < 0.0001.

8. The number of mice used in the supplementary fig. 4D and 4E is low. It is not clear how many times the experiment was repeated on different days. Power analysis should be performed, and I recommend using a biostatistical expert to determine this.

Response: Yes, thank you for the suggestion. We performed this experiment across two independent replicates, with each replicate including 2-3 mice. We have discussed this issue with a statistician and were suggested that the effect size of anakinra in mice *E.coli*

pneumonia is rather small compared with other experiments. To achieve a statistically significant P value, the sample size needs to expanded to at least 25-30 mice in each group, which state that anakinra is not such an effective drug for this disease. Additionally, we treated mice intraperitoneally with an anti-mIL-1β antibody at a widely used dose (200 μg each) (19-22) for IL-1β neutralization (Fig. S4F). Our findings indicated that neutralization of IL-1β did not attenuate *E.coli* pneumonia in the mice .

Fig. S4F

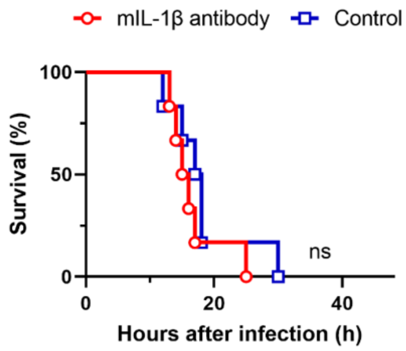

(S4F) WT mice were pretreated intratracheally with mIL-1β antibody/control antibody and subjected to lung *E.coli* infection. Survival was monitored for 48 hours. Statistical differences were determined by Mantel–Cox test. ns, not significant.

9. For the in vivo data, the authors often list the n number, which represents the number of used mice. However, whether the experiments were done on different days needs to be clarified.

Response: Yes, thank you for the suggestion. We have corrected this issue in the respective figure legend.

10. The authors should calculate and graph the intensity of bands from immunoblots in Fig. 2D and 2E.

Response: Yes, thank you for the suggestion. We additionally calculate the intensity of bands from the immunoblots shown in Fig. 2.

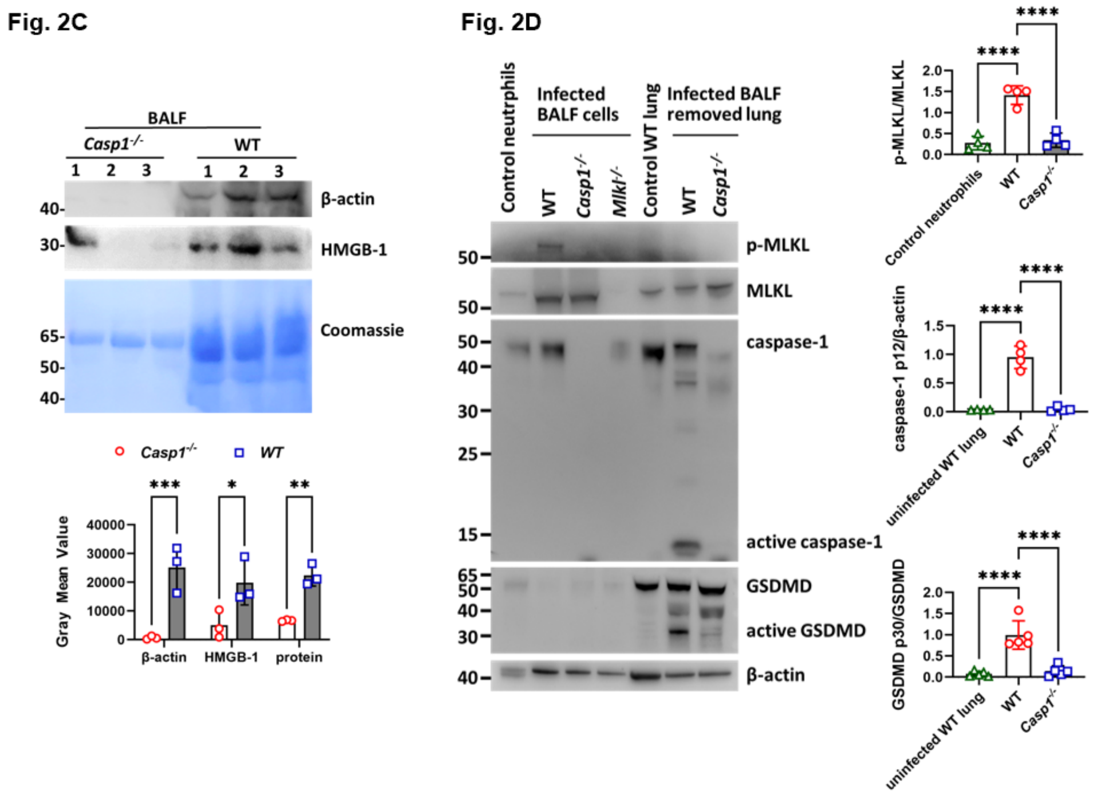

(Fig. 2C) HMGB1 levels in the BALF of *Casp1*$^{-/-}$ mice and WT mice at 12 hours after *E. coli* infection. Each lane represents one mouse (n = 3).

(Fig. 2D) BALF cells (containing > 95% neutrophils) and lung tissue without BALF cells from indicated mice were collected and immunoblotted for programmed cell death-related proteins.

Data are shown as the mean ± SD in (2C and 2D). Statistical differences were determined by the Student's *t* test (2C) and one-way ANOVA (2D). **P* < 0.05; ***P* < 0.01; ****P* < 0.001; *****P* < 0.0001.

11. What is the stain used in Fig. 4D? The authors should indicate detailed information in the figure legend and the method section.

Response: Yes, thank you for the suggestion. The stain used is PI, and we have indicated all detailed information in method section.

12. It is not stated how the anti-dsRNA was administered to mice.

Response: Yes, thank you for the suggestion. The anti-dsRNA antibody is administered intratracheally to the mice 1 hour before *E.coli* lung infection. We have included detailed information in both the legend and methods section.

13. It is not clear what is the purpose of measuring long terminal repeat elements (LTRs), long interspersed nuclear elements (LINEs), or short interspersed nuclear elements. The authors need to explain the rationale behind doing such an analysis. Is the dsRNA measured by RNAseq originate from epithelial or neutrophil?

Response: Yes, thank you for the question.

The purpose of measuring long terminal repeat elements (LTRs), long interspersed nuclear elements (LINEs), and short interspersed nuclear elements (SINEs) is to investigate the origin and potential role of dsRNA in the context of our study. By analyzing these repeat elements, we hope to gain insights into whether the dsRNA measured by RNA sequencing originates from specific genomic regions.

However, the dsRNA measured by RNAseq is derived from purified neutrophils

obtained from the BALF of infected mice, which is not the dsRNA that associated with *ZBP1*. Thus, it is not suitable to determine the possible dsRNA that promote cell death. We have removed this experiment from the revised manuscript.

14. The authors state "However, GW806742X is not a specific inhibitor of MLKL" What other proteins does the inhibitor target?

Response: This family of chemicals may also inhibit VEGFR2 (23). VEGFR is involved in both vasculogenesis and angiogenesis. Its activity is restricted mainly to cells of the vascular endothelium. VEGFR is widely associated with anti-cancer therapies.

Reference:

1. Kitur K, Parker D, Nieto P, Ahn DS, Cohen TS, Chung S, et al. Toxin-induced necroptosis is a major mechanism of Staphylococcus aureus lung damage. PLoS Pathog. 2015;11(4):e1004820.

2. González-Juarbe N, Gilley RP, Hinojosa CA, Bradley KM, Kamei A, Gao G, et al. Pore-Forming Toxins Induce Macrophage Necroptosis during Acute Bacterial Pneumonia. PLoS Pathog. 2015;11(12):e1005337.

3. de Bont CM, Boelens WC, Pruijn GJM. NETosis, complement, and coagulation: a triangular relationship. Cell Mol Immunol. 2019;16(1):19-27.

4. Alsabani M, Abrams ST, Cheng Z, Morton B, Lane S, Alosaimi S, et al. Reduction of NETosis by targeting CXCR1/2 reduces thrombosis, lung injury, and mortality in experimental human and murine sepsis. Br J Anaesth. 2022;128(2):283-93.

5. Liang W, Yang Y, Gong S, Wei M, Ma Y, Feng R, et al. Airway dysbiosis accelerates lung function decline in chronic obstructive pulmonary disease. Cell Host Microbe. 2023;31(6):1054-70.e9.

6. Moreira-Teixeira L, Stimpson PJ, Stavropoulos E, Hadebe S, Chakravarty P, Ioannou M, et al. Type I IFN exacerbates disease in tuberculosis-susceptible mice by inducing neutrophil-mediated lung inflammation and NETosis. Nat Commun. 2020;11(1):5566.

7. Wang Y, Cao C, Zhu Y, Fan H, Liu Q, Liu Y, et al. TREM2/β-catenin attenuates NLRP3 inflammasome-mediated macrophage pyroptosis to promote bacterial clearance of pyogenic bacteria. Cell Death Dis. 2022;13(9):771.

8. Zhang H, Du Y, Guo Y, Wang Z, Li H, Lv Z, et al. TLR4-NLRP3-GSDMD-Mediated Pyroptosis Plays an Important Role in Aggravated Liver Injury of CD38(-/-) Sepsis Mice. J Immunol Res. 2021;2021:6687555.

9. Yang S, Yang Y, Wang F, Luo Q, Zhang Y, Zheng F, et al. TREM2 Dictates Antibacterial Defense and Viability of Bone Marrow-derived Macrophages during Bacterial Infection. Am J Respir Cell Mol Biol. 2021;65(2):176-88.

10. Kelley N, Jeltema D, Duan Y, He Y. The NLRP3 Inflammasome: An Overview of Mechanisms of Activation and Regulation. Int J Mol Sci. 2019;20(13).

11. Bauer R, Rauch I. The NAIP/NLRC4 inflammasome in infection and pathology. Mol Aspects Med. 2020;76:100863.

12. Abban MK, Ayerakwa EA, Mosi L, Isawumi A. The burden of hospital acquired infections and antimicrobial resistance. Heliyon. 2023;9(10):e20561.

13. Bussini L, Pascale R, Rinaldi M, Bartoletti M. Diagnosis, management and treatment of nosocomial pneumonia in ICU: a narrative review. Journal of Emergency and Critical Care Medicine. 2022;6.

14. Kasetty G, Bhongir RKV, Papareddy P, Tufvesson E, Stenberg H, Bjermer L, et al. Osteopontin protects against pneumococcal infection in a murine model of allergic airway inflammation. Allergy. 2019;74(4):663-74.

15. Shi MM, Zhu YG, Yan JY, Rouby JJ, Summah H, Monsel A, et al. Role of miR-466 in mesenchymal stromal cell derived extracellular vesicles treating inoculation pneumonia caused by multidrug-resistant Pseudomonas aeruginosa. Clin Transl Med. 2021;11(1):e287.

16. Suri R, Periselneris J, Lanone S, Zeidler-Erdely PC, Melton G, Palmer KT, et al. Exposure to welding fumes and lower airway infection with Streptococcus pneumoniae. J Allergy Clin Immunol. 2016;137(2):527-34.e7.

17. Herold S, Tabar TS, Janssen H, Hoegner K, Cabanski M, Lewe-Schlosser P, et al. Exudate macrophages attenuate lung injury by the release of IL-1 receptor antagonist in gram-negative pneumonia. Am J Respir Crit Care Med. 2011;183(10):1380-90.

18. Iannitti RG, Napolioni V, Oikonomou V, De Luca A, Galosi C, Pariano M, et al. IL-1 receptor antagonist ameliorates inflammasome-dependent inflammation in murine and human cystic fibrosis. Nat Commun. 2016;7:10791.

19. Lawlor KE, Khan N, Mildenhall A, Gerlic M, Croker BA, D'Cruz AA, et al. RIPK3 promotes cell death and NLRP3 inflammasome activation in the absence of MLKL. Nat Commun. 2015;6:6282.

20. Copenhaver AM, Casson CN, Nguyen HT, Duda MM, Shin S. IL-1R signaling enables bystander cells to overcome bacterial blockade of host protein synthesis. Proc Natl Acad Sci U S A. 2015;112(24):7557-62.

21. Coffelt SB, Kersten K, Doornebal CW, Weiden J, Vrijland K, Hau CS, et al. IL-17-producing γδ T cells and neutrophils conspire to promote breast cancer metastasis. Nature. 2015;522(7556):345-8.

22. Hernández PP, Mahlakoiv T, Yang I, Schwierzeck V, Nguyen N, Guendel F, et al. Interferon-λ and interleukin 22 act synergistically for the induction of interferon-stimulated genes and control of rotavirus infection. Nat Immunol. 2015;16(7):698-707.

23. Sammond DM, Nailor KE, Veal JM, Nolte RT, Wang L, Knick VB, et al. Discovery

of a novel and potent series of dianilinopyrimidineurea and urea isostere inhibitors of VEGFR2 tyrosine kinase. Bioorg Med Chem Lett. 2005;15(15):3519-23.
