# Supplementary material for: Caspase-1-licensed pyroptosis drives dsRNA-mediated necroptosis and dampens host defense against bacterial pneumonia
Source: PLoS Pathog. 2025 May 13;21(5):e1013167. doi: 10.1371/journal.ppat.1013167 (PMC12121916; doi:10.1371/journal.ppat.1013167)
Supplement: Attachment — Submitted filename: Point to point response-chen-20250411.pdf [file ppat.1013167.s017.pdf]

Point to point response to the editor and reviewer

Part II – Major Issues: Key Experiments Required for Acceptance

Reviewer #3: 7) Histology scoring criteria were based on the severity of damaged lungs. How the damage was quantified remained unclear. The word "damage" is ambiguous for assessing disease severity.

Response: Thank you very much for this important methodological suggestion. For the histopathological evaluation, we have now clarified the scoring criteria in the revised manuscript: Assessment of histological lung injury was performed by grading as previous described and the details are as follows: 1, normal; 2, focal (<50% lung section) interstitial congestion and inflammatory cell infiltration; 3, diffuse (>50% lung section) interstitial congestion and inflammatory cell infiltration; 4, focal (<50% lung section) consolidation and inflammatory cell infiltration; 5, diffuse (>50% lung section) consolidation and inflammatory cell infiltration [47, 48]. For each section, at least five random high-power fields were selected for scoring. The mean score was used for comparison between groups."

Part III – Minor Issues: Editorial and Data Presentation Modifications

Reviewer #3:

1) Statements in lines 81-85 must be referenced.

Response: Yes, thanks you for the comments. Lines 81-85 now are referenced as follows: "Cohen et al. and Kebaier et al. reported that depletion of NLRP3 or inhibition of caspase-1 increased the bacterial killing ability of macrophages and conferred resistance to Staphylococcus aureus pneumonia in mice [10, 11]. Cohen et al. also reported that depletion of NLRC4 or inhibition of caspase-1 alleviated Pseudomonas aeruginosa pneumonia in mice [12]."

2) The statement in lines 152-154 "Although we observed more tissue-resident macrophages in the Casp1-/- mice in the physiological state, these cells almost completely disappeared at 12 hours after E. coli infection" is incorrect. Fig. 1D showed 10^4 macrophages at 12h, They have not completely disappeared as stated. Also, what did the authors mean by recruited macrophages? Macrophages are not recruited, monocytes do and then differentiate into macophages. Provide information in the figure legend on what markers were used to discriminate between different immune cells.

Response: We are sorry for the inaccuracy in our original description. The sentence has been revised in the manuscript as follows: "Although we observed more tissue-resident macrophages in the *Casp1*$^{-/-}$ mice in the physiological state, these macrophages decreased dramatically at 12 hours post-*E. coli* infection."

According to your suggestions, we also have changed "recruited macrophages" to "recruited monocytes". We have provided information of the markers used to discriminate between different immune cells in the figure legend as follows "Resident macrophages (SiglecF^{+}Ly6C^{-}F4/80^{+}) and recruited monocytes (SiglecF^{-}Ly6C^{+}F4/80^{+}) in the BALF of *Casp1*$^{-/-}$ mice (n = 11) and *Casp1*$^{+/+}$ mice (n = 8) at 12 hours after *E. coli* infection or intratracheal PBS instillation (n = 5 each) were counted by flow cytometry."

3) It is not clear how the RNAseq in Fig. S1J shows similar antimicrobial defense signals were induced by the two mouse strains (WT and Casp1 KO). There is only one set of data. What is the n=2 represent in there? How many mice per group were used for the RNA sequencing?

Response: Thanks for your concerns. To study the transcriptomic difference of *Casp1*$^{-/-}$ and *Casp1*$^{+/+}$ neutrophils, we isolate respective neutrophils from *E.coli* infected mice

(n = 2 biologically independent samples per genotype). RNA was extracted from these neutrophils and subjected to sequencing. Differentially expressed genes (DEGs) were identified by comparing these two genotypes (*Casp1*$^{-/-}$ and *Casp1*$^{+/+}$) using a parametric F test comparing nested linear models (*P* value < 0.05) and filtering results with stringent fold-change cutoffs (fold change > 2 or < 0.5). Then these DEGs were enriched by KEGG pathway analysis. Fig. S1J are the pathways that enriched from DEGs. n = 2 means 2 mice per group for RNA sequencing.

4) What cells are referred to in line 165?

Response: Thanks for your concerns. “These cells” referred to macrophages and neutrophils and we have modified in the revised manuscript as follows: “To further determine the role of neutrophils and macrophages in protecting against *E. coli*-induced pneumonia in *Casp1*$^{-/-}$ mice, we then used clodronate liposomes and an anti-Ly6G antibody to eliminate macrophages and neutrophils, respectively.”

5) Need a figure caption to the findings in lines 206-212.

Response: Thanks for your concerns. We have added the figure caption to lines 206-212 as follows: “We performed TUNEL staining for various cell markers in lung sections from the *Casp1*$^{+/+}$ and *Casp1*$^{-/-}$ mice (Fig. S3B). Our findings indicated that both *Casp1*$^{+/+}$ and *Casp1*$^{-/-}$ mice presented comparable TUNEL signals in macrophages (F4/80^{+}), with minimal signals observed in monocytes (CD14^{+}) and type II epithelial cells (SPC^{+}).”

6) For Fig. 2D, the authors must provide quantifications for all the bands, it is not clear why they chose to quantify only selected bands. If other bands are unnecessary, then

another blot should be provided.

Response: Yes, thank you very much for the comments. Actually, in Figure 2D, protein expression comparisons were limited to the same type of tissues (e.g., *Casp1*$^{+/+}$ BALF cells vs. *Casp1*$^{-/-}$ BALF cells). So we have adjusted the image so that all necessary quantifications were provided. All quantifications were normalized to β-actin or respective pro-protein within the same tissue, as annotated in the figure legends.

7) Change the double dose of bacteria in Fig. 3B to actual CFUs

Response: Yes, we have changed the double dose to actual CFUs in Fig. 3B.

8) Change "depletion" in line 254 to deletion.

Response: Yes, we are sorry for the mistake. We have changed "depletion" to "deletion" in line 254.

9) What is L929+T/Z in Fig. 4D? Please explain why this is done in the main text and needs to be mentioned in the figure legend.

Response: Yes, thank you for the suggestion. L929+T/Z means lysate of L929 cell line treated with TNF-α and Z-VAD, which is used as a positive control of p-RIPK3 for western blot. We have included this in the main text and figure legend.

10) Fig. 4F needs a better label to indicate that neutrophils were incubated with supernatants from indicated epithelial cell lines that were left uninfected (PBS) or infected (E.coli).

Response: Thanks for the good suggestions and we have changed a better label to indicate the treatment of neutrophils in Fig. 4F.

11) Fig. S8E was mentioned after Fig. S8F to H.

Response: Yes, we are sorry for the mistake. We have adjusted the manuscript to match the figures.

12) The authors concluded that administration of dsRNA antibody during E. coli infection strongly protected the mice in lines 375-376. However, the data (Fig. 5I) showed that the administration of dsRNA antibodies weakly protected mice from infection.

Response: Thanks for your concerns and we have changed the statement to match the conclusion as follows: "Moreover, administration of the dsRNA antibody significantly increased WT mice survival after *E.coli* infection."

13) There is no panel H in Fig. S2 as stated in the figure legend.

Response: We are sorry for this oversight and have adjusted the figure legend.

14) The quantification in Fig. S3B must be performed by One-way ANOVA.

Response: Thank you very much for the comments. In this experiment, our objective was to examine differences in TUNEL staining of cells within the lungs of mice with different genotypes. We focused on comparing differences in the same type of cells between the two genotypes, rather than comparing the difference among different types of cells. So we think that Student's *t*-test is statistically more appropriate for the comparisons.

15) The histology images in Fig. S5D, Fig. S8D, and Fig. S8E need to be labeled

Response: Thank you for the good suggestion. We are sorry for this oversight and have labeled Fig. S5D, Fig. S8D, and Fig. S8H.

16) Statistical analysis in Fig. S5 A, B, and C must be performed using Two-way ANOVA.

Response: Thanks for your correction and we have revised it.

17) What are the histograms shown in Fig. 7A? The x-axis needs to be labeled.

Response: Thanks for your reminder and the x-axis has been labeled in Fig. S7A.

18) Statistical analysis in Fig. S7D must be performed using Two-way ANOVA. All statistically significant data needs to be marked.

Response: Thanks for your concern. We have revised the statistical methods and there is no statistically significant data in this figure.

19) There should not be any statistical test performed on the image in Fig. 9A. Correct the figure legend.

Response: We are sorry for this oversight and we have corrected it.
