# Supplementary material for: Caspase-1-licensed pyroptosis drives dsRNA-mediated necroptosis and dampens host defense against bacterial pneumonia
Source: PLoS Pathog. 2025 May 13;21(5):e1013167. doi: 10.1371/journal.ppat.1013167 (PMC12121916; doi:10.1371/journal.ppat.1013167)
Supplement: Attachment — Submitted filename: Qinyu Luo et al manuscript review .docx [file ppat.1013167.s012.docx]

The manuscript by Qinyu Luo *et al* describes how epithelial cells-neutrophils crosstalk leading to the progression of pneumonia. Specifically, it showed during *E. coli* lung infection (pneumonia), Caspase1-mediated pyroptotic epithelial cells release dsRNA in the bronchoalveolar lavage fluid (BALF). As a result, dsRNA along with *E. coli* induce neutrophil necroptosis, which is associated with a detrimental impact on the disease outcome. The manuscript is well written except for the last portion of the result section. It used several genetic and pharmaceutical tools to support these findings. For the most part, I found the data supported the authors’ concussions. I think the findings are valuable to the field of lung immunopathology. The data will increase our understanding of how different death pathways are engaged in a complex environment with multiple types of cells to drive disease pathology. However, I have several concerns that could clear some of the ambiguous conclusions that the authors draw. The following are my concerns:

Major Concerns:

1. The presented data did not directly demonstrate *in vivo* that pyroptotic epithelial cells are responsible for dsRNA release in BALF. It is also not clear which cell death (Pyroptosis versus necroptosis) occurs first, or whether dead neutrophil causes epithelial cell damage and release of dsRNA. To address these, the authors should use epithelial cell-specific Caspase-1 deficient mice. However, generating such a mouse line will take at least 6 months. Instead, adoptive transfer experiment could provide some support to the conclusions. Bone marrow-derived neutrophils from wild-type mice could be adoptively transferred to UV-irradiated Caspase-1 KO mice and test pneumonia outcomes (mice survival; bacterial and immune cell counts; dsRNA level in the BALF and lungs; neutrophil and epithelial cell death and so on). The mice should be protected if the proposed conclusion is correct (i.e., pyroptotic epithelial cells released dsRNA to induce necroptotic neutrophils). Also, bone marrow-derived neutrophils from Caspase-1 KO mice could be adoptively transferred to UV-irradiated wild-type mice and test pneumonia outcomes. The experiment outcome could assess whether epithelial cell death or neutrophil death occurs first.
2. Which type of epithelial cells are affected? the authors should combine Tunel staining of histology sections from wild-type and Caspase1 KO with different cell markers including type I epithelial, type II epithelial, macrophages, neutrophils, and monocytes.
3. A combination of LPS + poly I:C stimulation to induce necroptotic neutrophils *in vitro* and *in vivo* should be tested. This will provide evidence that dsRNA from dying cells along with LPS from bacteria are sufficient to derive necroptotic neutrophils.
4. The authors need to measure bacterial loads in the lungs. Measuring bacterial counts in the BALF is not sufficient to conclude bacterial clearance. Bacterial counts in BALF depend on the efficiency of performing the lavage (do the authors use equal volume to lavage mice and whether end up with equal volumes of BALF from wild type and KOs. Also, immune cell counts in the lungs are a better predictor of disease outcomes than BALF.
5. Fig. 1D showed a massive loss of macrophage during infection. Are they dying? If so, which cell deaths are responsible, and could they contribute to dsRNA release? Why there are lower macrophage counts in uninfected wild-type relative to Caspase-1 KO? Do Caspase-1 KO mice experience some sort of low-grade inflammation, especially in the lungs?
6. All immune cells count, and bacterial burden should be graphed using a log y-axis scale. This way we could see the lower number of immune cells and bacteria that are present in some of the conditions.
7. It is not clear whether BALF cells in Fig. 2C or Fig. 4H that induced necroptosis are macrophages, monocytes, or neutrophils. Although neutrophil counts are higher in BALF, the amount of protein in these cells is very low compared to macrophages and monocytes. The authors should either sort these cells or magnetic purify them. Similarly, the remnant lung tissue after lavage is not only epithelial cells. Many other cells including interstitial macrophages could be the driver for activation of Caspase-1 and GSDMD.
8. Treated mice with control solvents such as DMSO, Saline, control IgG…etc are dying at a faster rate than untreated mice. Examples include in fig. 3A, fig. 3B, fig.5I, supplementary fig. 4D and 4E. Do the authors know why this is the case? Does DMSO or saline treatment make the mice more susceptible to infection? This should be emphasized.

Miner Concerns:

1. Impact neutrophil depletion on wild-type mice should be provided. Does neutrophil depletion impact bacterial clearance in wild-type mice?
2. The lung injury score graph (Fig. 1B) should be fixed back to a standard y-axis graph. It is not clear why a two-segment y-axis was used.
3. The last section of the results was poorly written (lines 325-381). There are a lot of missing spaces, the authors used “we next” several times to start a new sentence. It seems that the authors listing experiment findings without rationale or context.
4. In line 41 “conbined” should be fixed to combined.
5. The authors should immunofluorescence stain other BALF and lung cells with pMLKL antibody, like supplement fig. 2H. This could provide evidence that necroptosis impacted only neutrophils and/or other cells.
6. Does pyroptosis still occur in epithelial cells when the mice were treated with GW806742X or in Mlkl KO mice?
7. It is not clear how purified neutrophils are dying in response to *E. coli* and heat-killed *E. coli* in supplementary fig. 4A. The authors need to include more detail on how infections were performed. What is the multiplicity of infection (MOI) used and what time point post-infection cell death was analyzed? Are extracellular bacteria washed or kept in the media? Also, LPS-treated purified neutrophils induce activation of MLKL but no cell death. The authors need to explain why LPS stimulation is insufficient to trigger necroptosis.
8. The number of mice used in the supplementary fig. 4D and 4E is low. It is not clear how many times the experiment was repeated on different days. Power analysis should be performed, and I recommend using a biostatistical expert to determine this.
9. For the *in vivo* data, the authors often list the n number, which represents the number of used mice. However, whether the experiments were done on different days needs to be clarified.
10. The authors should calculate and graph the intensity of bands from immunoblots in Fig. 2D and 2E.
11. What is the stain used in Fig. 4D? The authors should indicate detailed information in the figure legend and the method section.
12. It is not stated how the anti-dsRNA was administered to mice.
13. It is not clear what is the purpose of measuring long terminal repeat elements (LTRs), long interspersed nuclear elements (LINEs), or short interspersed nuclear elements. The authors need to explain the rationale behind doing such an analysis. Is the dsRNA measured by RNAseq originate from epithelial or neutrophil?
14. The authors state “However, GW806742X is not a specific inhibitor of MLKL” What other proteins does the inhibitor target?
