# Supplementary material for: Caspase-1-licensed pyroptosis drives dsRNA-mediated necroptosis and dampens host defense against bacterial pneumonia
Source: PLoS Pathog. 2025 May 13;21(5):e1013167. doi: 10.1371/journal.ppat.1013167 (PMC12121916; doi:10.1371/journal.ppat.1013167)
Supplement: Attachment — Submitted filename: Qinyu Luo et al review comments.docx [file ppat.1013167.s014.docx]

The authors addressed some of the first round of reviews. However, there are still many concerns that need to be addressed. I also recommend having English as the first language colleague and working in the same field to edit the manuscript.

Major concerns:

1) The authors did not include the new data (TUNEL staining, stimulation with LPS+ poly I:C, and pMLKL immunofluorescence stain of BALF cells) in the revised manuscript. Only zoomed-out immunofluorescence images were provided, which made it difficult to analyze. The authors should provide higher-resolution zoomed-in images and quantify the data.

2) Measuring bacterial load in the lungs is still missing. Providing references that used BALF bacterial burden to assess lung pneumonia is weak. The authors should attempt at least to provide CFU data in the lungs of WT and Casp1 KO mice.

Minor concerns:

1) In the Abstract (line 18), what did the authors mean by “cell reaction”? Need some clarification.

2) In line 38, there is a missing period at the end of the sentence.

3) How is regulated cell death participating in the pathological process as stated in line 39? What do the authors mean by stating “regulated cell death”. Which cell death is not regulated? If cell death is controlled, then it should be beneficial.

4) The sentence in lines 248-253 is too long and needs to be broken into multiple sentences.

5) In lines 191-193, the authors included data regarding the clinical strain of bacteria, but there is no context. The authors should emphasize that the observed phenotype also occurs during infection with pathogenic E.coli. Information about the clinical isolate should be provided. I could not find the information in figure legends and materials/methods. Figures 2A and 2B should be labeled with the corresponding bacterial strain used to infect mice.

6) The authors need to indicate how they performed lung histology scoring.

7) Statistical analysis in Figures 1B, 1C, 1D, and 1E should be performed by two-way ANOVA.

8) In Fig.S2H, the authors need to provide zoomed images of neutrophils from Casp1 KO mice to compare to WT neutrophils.

9) In line 241. No need to start a new sentence.

10) In lines 243-245, the authors stated, “These findings also imply that caspase-1 may not act directly in the upstream of MLKL in this context, suggesting a potential alternative pathway in regulating neutrophil necroptosis during infection”. I am not following this train of thought. What data supports this conclusion?

11) No need for a new sentence in line 279.

12) In Figure S4E, treating mice with Anakinra is protective. The authors only used 6 mice, which may be insufficient to perform statistical analysis. A power analysis to identify sample size is needed. Provide the actual p-value for this graph.

13) The end of the paragraph in line 286 needs to have a conclusion sentence.

14) Two-way ANOVA must perform statistical analysis for figures 3C, 3D, and 3E.

15) Two-way ANOVA must perform statistical analysis for figures 4A, 4F, and 4G.

16) No need for a new sentence in line 320.

17) Two-way ANOVA must perform statistical analysis for figures S5B and S5C.

18) The sentence in lines 330-333 is too long and needs to be broken into multiple sentences.

19) No need for a new sentence in lines 341 and 345.

20) There is an extra period at the end of the sentence in line 342.

21) The end of the paragraph in line 350 needs to have a conclusion sentence.

22) No need for a new sentence in line 358.

23) Weirdly, poly(I:C) does not label control neutrophils in Figure 5G. How long the cells were stimulated?

24) The end of the paragraph in line 370 needs to have a conclusion sentence.

25) Two-way ANOVA must perform statistical analysis for figures 5F and 5H.

26) In Figure 6D, the authors indicated that they used WT control littermates. This should be labeled in the figures. Replace WT with either Zbp1+/+ or Zbp1+/- which is used in the experiment.

27) If control littermates were used in other figures as indicated in lines 464-466, the authors should replace them to indicate the mice genotype was used.

28) No need for a new sentence in lines 379 and 383.

29) Two-way ANOVA must perform statistical analysis for Figure 6E.

30) No need for a new sentence in lines 531 and 537.

31) No need for a new sentence in lines 558, 559, 620, and 642.
