# Supplementary material for: Caspase-1-licensed pyroptosis drives dsRNA-mediated necroptosis and dampens host defense against bacterial pneumonia
Source: PLoS Pathog. 2025 May 13;21(5):e1013167. doi: 10.1371/journal.ppat.1013167 (PMC12121916; doi:10.1371/journal.ppat.1013167)
Supplement: Attachment — Submitted filename: Response to Reviewers.docx [file ppat.1013167.s016.docx]

**Additional Editor Comments:**
I appreciate the effort that the authors gave to resolving the issues associated with this manuscript; however, there are still concerns that exist or remain unaddressed.

• I share a similar concern that bacterial burdens in BALF may not be reflective of whole tissue burdens, which was only addressed by citing a reference in the rebuttal letter. However, my larger concern is the lack of burden quantification in the in vivo models, in particular when quantifying in vivo functions. Burden quantifications are critical to ensure that the infection was consistent and that identified phenotypes are not associated with differences in inoculations or infection dynamics. Every in vivo characterization should be coupled with CFU burdens to ensure accuracy of the data.

Response: According to the editor’s suggestion, we performed bacterial CFU burdens for the whole lung tissue in infected mice in every major mechanistic point and present the results in the revised manuscript (Figure S1D, Figure S5E and Figure 6D).

(Figure S1D, Figure S5E and Figure 6D) Twelve hours after *E. coli* infection, the lungs were harvest from *Casp1*^-/-^ and *Casp1*^+/+^ (S1D), *Mlkl*^-/-^ and *Mlkl*^+/+^ (S5E), *Zbp1*^-/-^ and *Zbp1*^+/+^ (6D) mice, and bacterial burdens of the whole lung tissue were determined.

Data are shown as the mean ± SD. Statistical differences were determined by the Student’s *t* test (S5E, 6D) and two-way ANOVA (S1D). **P* < 0.05. CFU, colony-forming unit.

• Multiple pieces of data were provided in the rebuttal letter to address reviewer concerns, but these were not included in the manuscript (TUNEL staining with different cell populations, stimulation with LPS+ poly I:C, and pMLKL immunofluorescence stain of BALF cells). The point of these data is making the manuscript better not to directly address reviewers. This was an oversight not integrating these data into the manuscript. There were also instances in the text where conclusions are made without showing data (eg: line 161; phagocytosis efficiency…were comparable).

Response: We are sorry for this oversight and we have included these data in the revised manuscript (Figure S3B, Figure S4A, Figure 6F). In line 161, we have revised the conclusion as “We found that the ROS level in *Casp1*^-/-^ neutrophils were comparable to that in *Casp1^+/+^* neutrophils after *E. coli* challenge (Fig. S1I)”.

(Figure S3B) The lungs from *Casp1*^+/+^ and *Casp1^-/-^* mice were harvest at 12 hours post *E.coli* infection. TUNEL staining of lung sections with different cell markers as indicated was performed, and the count of TUNEL^+^ cells was quantified (*n* =  3). Scale bars, 50 μm.

(Figure S4A) The lungs from *Casp1*^+/+^ and *Casp1^-/-^* mice were harvest at 12 hours post *E.coli* infection. Immunofluorescence staining of lung sections with p-MLKL and neutrophil markers was performed.

(Figure 6F) WT neutrohpils were treated with control medium, LPS (1μg/ml) or LPS + poly(I:C) (1μg/ml) for 4 hours and cell death was determined by PI staining and flow cytometry analysis.

Data are presented as mean ± SD (S3B, 6F) and the images are representative of 3 independent experiments (S3B, S4A). Statistical differences were determined by the Student’s *t* test (S3B) or one-way ANOVA (6F). **P* < 0.05; ****P* <0.001. HPF, high-power field.

• The interpretation of Figure 5B is confusing. It seems unlikely that simply adding bacterial supernatant and MLE12 cell lysate nearly fully recreates the supernatant from pyroptotic MLE12 cells. These are entirely different stimulations. Also, if the stimulating agent is dsRNA that gets released from the epithelial cells, does pyroptosis cause an abundance of dsRNA? It is unclear why dsRNA (which would likely be in low abundance) would be such a potent stimulation when the abundance of cytokines and other nuclear molecules (dsDNA, ssRNA) would be much higher. In addition, why would dsRNA only be found in the BALF, but not identified in the lung homogenates (Fig. 5E)?

Response: Thanks for your concerns. We acknowledge that the combination of bacterial supernatant and MLE12 cell lysate cannot fully equate with pyroptotic MLE12 supernatant. However, our findings demonstrate that this combination significantly accelerates neutrophil death compared to either component alone, suggesting that bacterial secretions and intracellular host factors act synergistically to induce necroptosis (Figure 4B). While this does not fully replicate the pyroptotic supernatant, it highlights the cooperative role of bacterial and host-derived signals in driving neutrophil death. We have modified the main text in line 305 as follows: “A substantial amount of neutrophil death was induced by the pyroptotic supernatant from live *E. coli*-stimulated MLE-12 cells (Fig. 4B), whereas neither the supernatant from the cell lysate nor that from cultured *E. coli* alone induced neutrophil death. Interestingly, although these cannot be fully equated, combining the two supernatants resulted in significant neutrophil death (Fig. 4B).”

Regarding the role of dsRNA, we observed that pyroptotic MLE12 cells release substantial amounts of dsRNA, which is also present in MLE12 cell lysates (Figure 5D). Neutralization experiments confirmed the necessity of dsRNA, as blocking dsRNA significantly reduced neutrophil death induced by pyroptotic supernatant. Given that ZBP1, a key mediator of necroptosis, can be activated by dsRNAs (1-3), we focused on dsRNA rather than other nucleic acids. As for the detection of dsRNA in BALF versus lung homogenates (Figure 5E), dsRNA was present in both, but the lower signal intensity in lung homogenates is due to differences in sample dilution and exposure time during processing, not the absence of dsRNA. We have replaced this image with another replicate. These findings collectively underscore the importance of dsRNA as a potent stimulator of neutrophil necroptosis in this context.

(Figure 4B) Neutrophil death after stimulation with MLE12 lysate supernatant (MLE12 lysate sup), *E. coli* culture supernatant (Bac. sup) and the mixture of these supernatants, and pyroptotic supernatant (pyrop. sup) as well as control medium (n = 3).

(Figure 5D) Dot blot of MLE12 lysate supernatant (MLE12 lysate sup), *E. coli* culture supernatant (Bac. sup) and pyroptotic supernatant (pyrop. sup) as well as control medium using dsRNA antibody.

(Figure 5E) Dot blot of BALF from indicated chimeric mice at 12 hours after pulmonary *E. coli* infection using anti-dsRNA antibody.

Data are shown as the mean ± SD in (4B) and are representative of 3 independent experiments in (5D, 5E). Statistical differences were determined by one-way ANOVA (4B). **P* < 0.05; *****P* < 0.0001.

• The authors use an antibody to neutralize dsRNA during infection (Fig. 5I-K), but rather than simply neutralizing the dsRNA, this likely created immune complexes and would skew the inflammatory environment. It's really uncertain what the dsRNA antibody is doing, especially in vivo. A cleaner experiment would be to use TLR3-deficient mice, which are readily available, and prevent signaling by dsRNA. TLR3 seems the most likely receptor mediating neutrophil necroptosis and it is unusual that this was overlooked in the manuscript.

Response: Thank you for your suggestion. Indeed, TLR3 is a known dsRNA receptor. As shown by RNA sequencing data in Figure 5A and western blot analysis in Supplementary Figure S9A, neutrophils from these mice do not express TLR3. We further used TLR3-deficient mice to conduct the *in vivo* experiment. We found that TLR3 deficiency did not prevent neutrophil death and decrease bacteria burden (Figure S9, B-E). Thus this experiment should help clarify the role of dsRNA *in vivo*.

(Figure 5A) RNA sequencing of gene expression of neutrophils in the BALF of *Casp1^-/-^* mice and *Casp1^+/+^* mice at 12 hours after pulmonary *E. coli* infection. FPKM, fragments per kilobase per million mapped fragments. n = 2 for each group.

(Figure S9A) Western blot analysis of BALF neutrophils and spleen tissue (as positive control) derived from *E.coli* infected *Casp1*^-/-^ and *Casp1*^+/+^ mice at 12 hours post infection.

(Figure S9, B-E) Twelve hours after *E. coli* infection, the proportion of PI^+^ neutrophils (A), neutrophil numbers (B), total leukocytes (C) in BALF and bacterial burdens in lung tissue (D) from *Tlr3*^+/+^ mice (n = 8) and *Tlr3^-/-^* mice (n = 7) were determined.

Data are shown as the mean ± SD (S9, B-E) and are representative of 2 independent experiments in S9A. Statistical differences were determined by the Student’s *t* test (S9, A-D). ns, not significant. CFU, colony-forming unit.

Data quality concerns:
• In Figure 5B the input lane is bands while the IP lanes look highly smeared or dot blot-like (ZBP1 bands). Presumably this was all run on the same gel, so why are the lanes so variable in presentation?

Response: Thank you very much for the concern. This may be due to some technical problems. We redo this experiment and the bands seems to be normal (Figure 5B). We replace this image in the revised manuscript.

(Figure 5B) Immunoprecipitation (IP) assay using anti-ZBP1 antibody or IgG control antibody in BALF cells from WT mice at 12 hours after pulmonary *E. coli* infection. Data are representative of 3 independent experiments.

• The fluorescent images provided in Figure 5G are not of sufficient quality to draw any conclusions. The pattern of the dapi stain seems unusual and doesn’t seem to be staining the nucleus. The background of the Poly(I:C) in the bacterial stimulated cells looks excessive and localized to a specific region, which is not present in the mock stimulated. Despite these concerns in the images, providing a single cell is not sufficient to draw conclusions. Higher quality representative images and quantification is required.

Response: According to the editor’s suggestions, we revised these images and added the quantification data into Figure 5G.

(Figure 5G) Representative immunofluorescence images of neutrophils cocultured with control/Bac.sup and FITC-conjugated poly(I:C) using PKH26 and anti-ZBP1 antibody and quantification of the percentage of AF647/FITC colocalized cells (*n* =  3 ). Scale bars, 10 μm.

Data are mean ± SD and are representative of 3 independent experiments. Statistical differences were determined by the Student’s *t* test. ***P* < 0.01.

• Supplementary Figure 2I includes images of BALF cells with high pMLKL protein at the membrane. Based on the proposed model, the neutrophils are the cell type with high pMLKL protein; however, the nuclear staining (Dapi) of these cells do not look neutrophil-like. This is concerning and requires further validation (Ly6g or neutrophil specific protein staining) to ensure the cell type of the BALF cells.

Response: According to the editor’s suggestion, we stained Ly6G to BALF cells to ensure the cell type of the BALF cells in Figure S4B (Figure S2I in original script).

(Figure S4B) BALF cells from *Casp1^-/-^* mice and *Casp1^+/+^* mice were fixed on slides, stained with PKH26, Ly6G and p-MLKL antibodies and visualized by using confocal microscopy. Scale bars, 10 μm. Data are representative of 3 independent experiments.

**Reviewers' Comments:**
**Part I - Summary**
Reviewer #3: The authors addressed some of the first round of reviews. However, there are still many concerns that need to be addressed. I also recommend having English as the first language colleague and working in the same field to edit the manuscript.

Response: Thank you very much for your concern. The manuscript has been edited by a native English-speaking colleague to ensure linguistic accuracy.

**Part II – Major Issues: Key Experiments Required for Acceptance**

Reviewer #3: 1) The authors did not include the new data (TUNEL staining, stimulation with LPS+ poly I:C, and pMLKL immunofluorescence stain of BALF cells) in the revised manuscript. Only zoomed-out immunofluorescence images were provided, which made it difficult to analyze. The authors should provide higher-resolution zoomed-in images and quantify the data.

Response: According to the reviewer’s suggestion, we have included these data in revised manuscript as Figure S3B, Figure S4A and Figure 6F and provide zoomed-in images and quantification of the data.

(Figure S3B) The lungs from *Casp1*^+/+^ and *Casp1^-/-^* mice were harvest at 12 hours post *E.coli* infection. TUNEL staining of lung sections with different cell markers as indicated was performed, and the count of TUNEL^+^ cells was quantified (*n* =  3). Scale bars, 50 μm.

(Figure S4A) The lungs from *Casp1*^+/+^ and *Casp1^-/-^* mice were harvest at 12 hours post *E.coli* infection. Immunofluorescence staining of lung sections with p-MLKL and neutrophil markers was performed.

(Figure 6F) WT neutrohpils were treated with control medium, LPS (1μg/ml) or LPS+poly(I:C) (1μg/ml) for 4 hours and neutrophil death was determined by PI staining and flow cytometry analysis.

Data are presented as mean ± SD (S3B, 6F) and the images are representative of 3 independent experiments (S3B, S4A). Statistical differences were determined by the Student’s *t* test (S3B) or one-way ANOVA (6F). **P* < 0.05; ****P* <0.001. HPF, high-power field.

2) Measuring bacterial load in the lungs is still missing. Providing references that used BALF bacterial burden to assess lung pneumonia is weak. The authors should attempt at least to provide CFU data in the lungs of WT and Casp1 KO mice.

Response: According to the reviewer’s suggestion, we performed bacterial load in the lung tissue in major mechanistic point such as Figure S1D, Figure S5E and Figure 6D.

(Figure S1D, Figure S5E and Figure 6D) Twelve hours after *E. coli* infection, the lungs were harvest from *Casp1*^-/-^ and *Casp1*^+/+^ (S1D), *Mlkl*^-/-^ and *Mlkl*^+/+^ (S5E), and *Zbp1*^-/-^ and *Zbp1*^+/+^ (6D) mice, and bacteria burdens of the whole lung tissue were determined.

Data are shown as the mean ± SD. Statistical differences were determined by the Student’s *t* test (S5E, 6D) and two-way ANOVA (S1D). **P* < 0.05. CFU, colony-forming unit.

**Part III – Minor Issues: Editorial and Data Presentation Modifications**
Reviewer #1: The authors should quantify results shown in figures Fig. 5F and 5G.

Response: Yes, thank you for the suggestion. We have quantified the results shown in figures Fig. 5F and 5G.

(Figure 5F) Neutrophil interaction of FITC-conjugated poly(I:C) and neutrophil death (PI^+^) were measured by flow cytometry after stimulation with *E. coli* culture supernatant (Bac. Sup) or control medium with/without FITC-conjugated poly(I:C) (n = 3).

(Figure 5G) Representative immunofluorescence images of neutrophils cocultured with control/Bac.sup and FITC-conjugated poly(I:C) using PKH26 and anti-ZBP1 antibody and quantification of the percentage of AF647/FITC colocalized cells (*n*  =  3). Scale bars, 10 μm.

Data are presented as the mean ± SD and the images are representative of 3 independent experiments. Statistical differences were determined by the Student’s *t* test. ***P* < 0.01.

Reviewer #3: 1) In the Abstract (line 18), what did the authors mean by “cell reaction”? Need some clarification.

Response: Yes, thank you for the comment. The term "cell reaction" has been included within "host response" to more accurately and concisely describe the observed in vivo and in vitro experiments. We have adjusted this as: “Bacterial lung infections cause severe host responses.”

2) In line 38, there is a missing period at the end of the sentence.

Response: Thanks for your kindness and we have corrected this mistake.

3) How is regulated cell death participating in the pathological process as stated in line 39? What do the authors mean by stating “regulated cell death”. Which cell death is not regulated? If cell death is controlled, then it should be beneficial.

Response: Yes, thank you for the comment. The term "regulated cell death" typically refers to a form of cell death that is controlled by specific molecular pathways and is often considered a part of immune responses. This contrasts with "unregulated" cell death, which can occur due to physical damage or injury, leading to necrosis, where cells die in an uncontrolled manner.

Regulated cell death refers to a process in which the cell regulates its own death through intrinsic molecular pathways rather than the host-controlled cell death. These pathways can be triggered by cell extrinsic damage-associated molecular patterns (DAMPs), pathogen-associated molecular patterns (PAMPs), or other stimuli, and their activation can be inhibited by the depletion of cell intrinsic pathways.

While regulated cell death is beneficial in some contexts (e.g., clearing damage cells), dysregulation or excessive activation of these processes can contribute to disease progression (4-6).

4) The sentence in lines 248-253 is too long and needs to be broken into multiple sentences.

Response: Yes, thanks for your suggestion. We have corrected this sentence as follows:

“Recent studies have shown that certain pathogens can trigger the formation of the PANoptosome, a multiprotein complex. This complex is composed of caspase-1, caspase-8, RIPK1, RIPK3, Fas-associated protein with death domain, apoptosis-related spot-like protein, AIM2, pyrin, and ZBP1. The PANoptosome induces PANoptosis, a process that includes apoptosis, pyroptosis, and necroptosis”

5) In lines 191-193, the authors included data regarding the clinical strain of bacteria, but there is no context. The authors should emphasize that the observed phenotype also occurs during infection with pathogenic E.coli. Information about the clinical isolate should be provided. I could not find the information in figure legends and materials/methods. Figures 2A and 2B should be labeled with the corresponding bacterial strain used to infect mice.

Response: Yes, thanks for your suggestion. We add the context and observation of the clinical-isolated *E. coli* in line 195 as “A pathogenic bacterial strain is crucial in the study of human disease, as it closely mimics the pathogens responsible for human infection. Thus, we include a clinical-isolated *E.coli* strain *in vivo*. Likely, we observed lower neutrophil death with this pathogenic *E.coli.*”

Also, we provided the information about this clinical isolate in materials as “The clinical strain of *E. coli* was isolated from a hospitalized patient with septic arthritis in the knee at the Children’s Hospital, Zhejiang University School of Medicine, Hangzhou, China*.*” The figures are also revised as followed (Figure 2A and 2B).

(Figure 2A and 2B) Death of neutrophils in the BALF of *Casp1^-/-^* mice and *Casp1^+/+^* mice at 12 hours after *E. coli* ATCC25922 (n = 6) or clinical isolated *E. coli* strain (n = 8-10) infection. Cell viability was measured with PI staining and analyzed using flow cytometry.

Data are shown as the mean ± SD. Statistical differences were determined by the Student’s *t* test. ***P* < 0.01.

6) The authors need to indicate how they performed lung histology scoring.

Response: Yes, thanks for your suggestion. We have included this information in materials and methods in line 569: “Histopathological features were scored on a 5-point scale: 0, minimal damage; 1–2, mild damage; 2–3, moderate damage; 3–4, severe damage; and 4+, maximal damage. Two examiners performed histopathological analysis without prior knowledge of the experimental interventions (7). ”

7) Statistical analysis in Figures 1B, 1C, 1D, and 1E should be performed by two-way ANOVA.

Response: Yes, thanks for your suggestion. We have performed statistical analysis in Figures 1B, 1C, 1D, and 1E by two-way ANOVA.

8) In Fig.S2H, the authors need to provide zoomed images of neutrophils from Casp1 KO mice to compare to WT neutrophils.

Response: Yes, thanks for the suggestions. We provide zoomed images of neutrophils from casp1 KO mice (Figure S4B) in the revised manuscript.

(Figure S4B) BALF cells from *Casp1^-/-^* and *Casp1^+/+^* mice were fixed on slides, stained with PKH26, Ly6G and p-MLKL antibodies and visualized by using confocal microscopy. Scale bars, 10 μm. Data are representative of 3 independent experiments.

9) In line 241. No need to start a new sentence.

Response: Yes, thanks for your kindly correction. We have corrected it.

10) In lines 243-245, the authors stated, “These findings also imply that caspase-1 may not act directly in the upstream of MLKL in this context, suggesting a potential alternative pathway in regulating neutrophil necroptosis during infection”. I am not following this train of thought. What data supports this conclusion?
Response: Thanks for your concern to this issue, our conclusion that caspase-1 may not act directly upstream of MLKL in this context is supported by data presented in Figure 3B. We observed that treatment with an MLKL inhibitor in *Casp1* knockout mice infected with a double dose of *E. coli* resulted in mildly improved outcomes. If caspase-1 were strictly required for MLKL activation during infection, the MLKL inhibitor would not be expected to have any additional effect in *Casp1* knockout mice. The fact that MLKL inhibition provided further protection in these mice suggests the existence of an alternative pathway regulating neutrophil necroptosis that operates independently of caspase-1 in some certain situations. We have adjusted this sentence to line 245 as: “a mild benefit was observed when infected with twice dose of *E. coli* (Fig. 3B), suggesting a potential alternative pathway independent of caspase-1 in regulating neutrophil necroptosis.”

(Figure 3B) Survival of *Casp1^-/-^* mice treated with the MLKL inhibitor GW806742X (n = 7) or control solvent (n = 8) after pulmonary *E. coli* infection with a single dose of *E. coli*, and survival of *Casp1^-/-^* mice treated with the MLKL inhibitor GW806742X (n = 8) or control solvent (n = 7) after pulmonary infection with a double dose of *E. coli*. This experiment was conducted across three independent replicates, with each replicate including 2-3 mice.

Statistical differences were determined by the Mantel‒Cox test. **P* < 0.05. ns, not significant.

11) No need for a new sentence in line 279.
Response: Thanks for your kindly suggestion, and we have made the adjustment.

12) In Figure S4E, treating mice with Anakinra is protective. The authors only used 6 mice, which may be insufficient to perform statistical analysis. A power analysis to identify sample size is needed. Provide the actual p-value for this graph.
Response: Yes, thank you very much for the comment. We additionally used 7 mice in each group to supplement this experiment and found that Anakinra treatment did not reverse the survival rate of these infected WT mice (Figure S5E). The p-value (n = 13) is 0.7696 now.

(Figure S5E) Survival of WT mice intratracheally (n = 13 each) pretreated with the IL-1β antagonist anakinra or saline after pulmonary *E. coli* infection. Statistical differences were determined by the Mantel‒Cox test. ns, not significant.

13) The end of the paragraph in line 286 needs to have a conclusion sentence.
Response: Yes, thanks for your kindly correction. We have revised as follows: “These results indicated that these nonhematopoietic cells are inflammasome-activated cells during lung *E. coli* infection.”

14) Two-way ANOVA must perform statistical analysis for figures 3C, 3D, and 3E.
Response: Yes, thanks for your suggestion. We have performed statistical analysis in Figures 3C, 3D, and 3E by two-way ANOVA.

15) Two-way ANOVA must perform statistical analysis for figures 4A, 4F, and 4G.
Response: Yes, thanks for your suggestion. We have performed statistical analysis in Figures 4A, 4F and 4G by two-way ANOVA.

16) No need for a new sentence in line 320.
Response: Yes, thanks for your kindly correction and we have revised it.

17) Two-way ANOVA must perform statistical analysis for figures S5B and S5C.
Response: Yes, thanks for your suggestion. We have performed statistical analysis in Figures S5B and S5C by two-way ANOVA.

18) The sentence in lines 330-333 is too long and needs to be broken into multiple sentences.
Response: Yes, thanks for your kindly correction. We have revised the sentence as follows: “During necroptosis, RIPK3 is phosphorylated and activated by proteins containing the RIP homotypic interaction motif (RHIM). These RHIM-containing proteins include TRIF and ZBP1. Additionally, RIPK1, another RHIM-containing protein, contributes to RIPK3 activation”

19) No need for a new sentence in lines 341 and 345.
Response: Yes, thanks for your kindly correction and we have revised it.

20) There is an extra period at the end of the sentence in line 342.
Response: Thanks for your kindly correction for this mistake.

21) The end of the paragraph in line 350 needs to have a conclusion sentence.
Response: Yes, thanks for your kindly correction. We have revised as follows: “These results demonstrate that nonhematopoietic pyroptosis plays a critical role in the release of dsRNA.”

22) No need for a new sentence in line 358.
Response: Yes, thanks for your kindly correction and we have revised it.

23) Weirdly, poly(I:C) does not label control neutrophils in Figure 5G. How long the cells were stimulated?
Response: Thanks for your concerns. Based on our result from Figure 5F, the co-stimulation of Bac.sup did increase the interaction of poly(I:C) to neutrophils. However, there is still some poly(I:C) that entered control cells. We have adjusted the figures. The cells were stimulated for 4 hours and we have included the detailed information in material section as “For immunofluorescence analysis, purified neutrophils were treated with LPS (1 μg/ml) and poly(I:C) (1 μg/ml) for 4 hours, and the cells were collected, stained with PKH26 and distributed on slides. After drying, fixation, and permeabilization, the cells were stained with anti-ZBP1 and anti-AF647 secondary antibodies (MedChemExpress) for analysis. Images were taken with an Olympus FV3000 microscope (Olympus), and the data were analyzed with ImageJ software (NIH).”

(Figure 5F) Neutrophil interaction of FITC-conjugated poly(I:C) and neutrophil death (PI^+^) were measured by flow cytometry after stimulation with *E. coli* culture supernatant (Bac. Sup) or control medium with/without FITC-conjugated poly(I:C) (n = 3).

(Figure 5G) Representative immunofluorescence images of neutrophils cocultured with control/Bac.sup and FITC-conjugated poly(I:C) using PKH26 and anti-ZBP1 antibody and quantification of the percentage of AF647/FITC colocalized cells (*n* =  3). Scale bars, 10 μm.

Data are presented as the mean ± SD and the images are representative of 3 independent experiments. Statistical differences were determined by the Student’s *t* test. ***P* < 0.01.

24) The end of the paragraph in line 370 needs to have a conclusion sentence.
Response: Yes, thanks for your kindly correction. We have revised as follows: “These results indicate that dsRNA neutralization ameliorates lung injury and promotes bacterial clearance by reducing neutrophil death and inflammation, but its protective effects depend on intact caspase-1-, MLKL-, and ZBP1-mediated pathways.”

25) Two-way ANOVA must perform statistical analysis for figures 5F and 5H.
Response: Yes, thanks for your suggestion. We have performed statistical analysis in Figures 5F and 5H by two-way ANOVA.

26) In Figure 6D, the authors indicated that they used WT control littermates. This should be labeled in the figures. Replace WT with either Zbp1+/+ or Zbp1+/- which is used in the experiment.
Response: Thanks for your correction. We have replaced these “WT” mice with indicated genotypes.

27) If control littermates were used in figures as indicated in lines 464-466, the authors should replace "WT" to indicate the WT mice genotype (i.e., Casp1+/+ or Casp1+/-).
Response: Thanks for your correction. We have replaced these “WT” mice with indicated genotypes.

28) No need for a new sentence in lines 379 and 383.
Response: Yes, thanks for your kindly correction and we have revised it.

29) Two-way ANOVA must perform statistical analysis for Figure 6E.
Response: Yes, thanks for your suggestion. We have performed statistical analysis in Figures 6E by two-way ANOVA.

30) No need for a new sentence in lines 531 and 537.
Response: Yes, thanks for your kindly correction and we have revised it.

31) No need for a new sentence in lines 558, 559, 620, and 642.

Response: Yes, thanks for your kindly correction and we have revised it.

Reference:

1.	Jiao H, Wachsmuth L, Kumari S, Schwarzer R, Lin J, Eren RO, et al. Z-nucleic-acid sensing triggers ZBP1-dependent necroptosis and inflammation. Nature. 2020;580(7803):391-5.

2.	Kuriakose T, Man SM, Malireddi RK, Karki R, Kesavardhana S, Place DE, et al. ZBP1/DAI is an innate sensor of influenza virus triggering the NLRP3 inflammasome and programmed cell death pathways. Sci Immunol. 2016;1(2).

3.	Upton JW, Kaiser WJ, Mocarski ES. DAI/ZBP1/DLM-1 complexes with RIP3 to mediate virus-induced programmed necrosis that is targeted by murine cytomegalovirus vIRA. Cell Host Microbe. 2012;11(3):290-7.

4.	Linkermann A, Green DR. Necroptosis. N Engl J Med. 2014;370(5):455-65.

5.	Man SM, Karki R, Kanneganti TD. Molecular mechanisms and functions of pyroptosis, inflammatory caspases and inflammasomes in infectious diseases. Immunol Rev. 2017;277(1):61-75.

6.	Vandenabeele P, Galluzzi L, Vanden Berghe T, Kroemer G. Molecular mechanisms of necroptosis: an ordered cellular explosion. Nat Rev Mol Cell Biol. 2010;11(10):700-14.

7.	Wang H, Zeng C, Luo G, Sun Y, Zhang J, Xu Z, et al. Macrophage ferroportin serves as a therapeutic target against bacteria-induced acute lung injury by promoting barrier restoration. iScience. 2022;25(12):105698.
